# Regulation of invasion and peritoneal dissemination of ovarian cancer by mesothelin manipulation

**DOI:** 10.1038/s41389-020-00246-2

**Published:** 2020-07-01

**Authors:** Ricardo Coelho, Sara Ricardo, Ana Luísa Amaral, Yen-Lin Huang, Mariana Nunes, José Pedro Neves, Nuno Mendes, Mónica Nuñez López, Carla Bartosch, Verónica Ferreira, Raquel Portugal, José Manuel Lopes, Raquel Almeida, Viola Heinzelmann-Schwarz, Francis Jacob, Leonor David

**Affiliations:** 1 Differentiation and Cancer group, Institute for Research and Innovation in Health (i3S), University of Porto, Porto, Portugal; 2 Institute of Molecular Pathology and Immunology of the University of Porto (IPATIMUP), Porto, Portugal; 3 Faculty of Medicine, University of Porto, Porto, Portugal; 4 Glyco-Oncology, Ovarian Cancer Research, University Hospital Basel and University of Basel, Basel, Switzerland; 5 ICBAS, Institute of Biomedical Sciences Abel Salazar, University of Porto, Porto, Portugal; 6 Pathology Department, Centro Hospitalar de São João, Porto, Portugal; 7 Histology and Electron Microscopy, Institute for Research and Innovation in Health (i3S), University of Porto, Porto, Portugal; 8 Department of Pathology, Portuguese Oncology Institute of Porto (IPO-Porto), Porto, Portugal; 9 Cancer Cell Signaling and Metabolism Group, Institute for Research and Innovation in Health (i3S), University of Porto, Porto, Portugal; 10 Biology Department, Faculty of Sciences of the University of Porto, Porto, Portugal; 11 Gynecological Cancer Center and Ovarian Cancer Research, Department of Biomedicine, University Hospital Basel and University of Basel, Basel, Switzerland

**Keywords:** Ovarian cancer, Cancer models

## Abstract

Peritoneal dissemination is a particular form of metastasis typically observed in ovarian cancer and the major cause for poor patient’s outcome. Identification of the molecular players involved in ovarian cancer dissemination can offer an approach to develop treatment strategies to improve clinical prognosis. Here, we identified mesothelin (MSLN) as a crucial protein in the multistep process of peritoneal dissemination of ovarian cancer. We demonstrated that MSLN is overexpressed in primary and matched peritoneal metastasis of high-grade serous carcinomas (HGSC). Using several genetically engineered ovarian cancer cell lines, resulting in loss or gain of function, we found that MSLN increased cell survival in suspension and invasion of tumor cells through the mesothelial cell layer in vitro. Intraperitoneal xenografts established with MSLN^high^ ovarian cancer cell lines showed enhanced tumor burden and spread within the peritoneal cavity. These findings provide strong evidences that MSLN is a key player in ovarian cancer progression by triggering peritoneal dissemination and provide support for further clinical investigation of MSLN as a therapeutic target in HGSC.

## Introduction

Peritoneal dissemination is a particular form of metastasis in ovarian^1^, pancreatic^2^ and gastrointestinal cancers^3^. In this multistep process of cancer dissemination, cancer cells detach from primary tumor, survive and circulate in the peritoneal fluid and implant through the mesothelial layer with subsequent peritoneal carcinomatosis^4,5^. The presence of peritoneal carcinomatosis is usually associated with accumulation of ascitic fluid and an aggressive disease course and poor prognosis^6^. Specifically, in ovarian cancer this form of malignant progression is considered to be the most common route for metastasis^1^. Elucidation of the molecular players involved in the peritoneal dissemination can provide further insights into the development of therapeutic approaches to improve clinical outcomes of ovarian cancer patients.

MSLN is overexpressed in several human malignancies that display a strong predilection for the peritoneal cavity as the site of metastasis such as ovarian cancer^7,8^, pancreatic cancer^9^, and gastric cancer^10^. First identified in 1992^11^, MSLN is synthesized as a 70 kDa precursor protein that is proteolytically cleaved by Furin, resulting in a soluble 30 kDa fragment called megakaryocyte potentiation factor (MPF) and the 40 kDa MSLN membrane bound fragment^8^. Although the physiological function of MSLN, as well as its role in cancer, are still unclear, it was originally suggested that MSLN could have a role in cell adhesion^8^. The role in the adhesion process was further supported by evidence showing that binding of MSLN to mucin MUC16 could be important for the peritoneal homing of ovarian cancer^12^. In addition, several studies have suggested a function for MSLN in cell survival, invasion, tumor progression, and chemoresistance^13–16^. However, the relevance of MSLN expression in the peritoneal dissemination of ovarian cancer cells has not yet been addressed. Therefore, unveiling the role of MSLN in the malignant progression of ovarian cancer will clarify its possible interest in clinical applications, including prognostic evaluation and therapeutic targeting.

Here, we found that high MSLN expression levels are associated with the presence of ascites and shorter progression-free survival in epithelial ovarian cancer (EOC) patients and that primary and matched peritoneal metastasis of HGSC share MSLN overexpression. Through in vitro and in vivo studies, we found that MSLN expression contributes to the uniqueness of ovarian cancer dissemination in the peritoneum by promoting cell survival in suspension, invasion through the mesothelial layer and spread within the peritoneal cavity. Collectively, these results provide strong evidences that MSLN is a key player in the multistep process of peritoneal dissemination in ovarian cancer.

## Results

### MSLN expression levels have an impact on ovarian cancer (particularly in HGSC) behavior, and *MSLN* edited models were built to explore this outcome

**Fig. 1:**
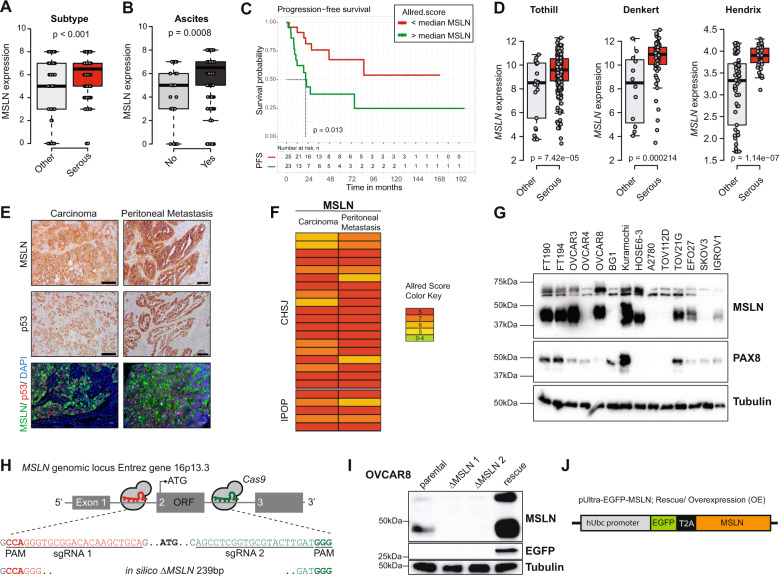
MSLN expression levels are associated with poor prognosis of ovarian cancer, and development of *MSLN* edited models to explore this outcome. **a** Boxplot showing a significantly higher MSLN expression in EOC of the serous subtypes (*n* = 124 cores) compared with other subtypes (*n* = 102 cores) for 48 EOC patients, *p* value calculated by Student’s *t* test. **b** Boxplot showing a significant association between the higher levels of MSLN expression and the presence of ascites at primary diagnosis in EOC patients; No, *n* = 32 cores; Yes, *n* = 124 cores; *p* value calculated by Student’s *t* test. **c** Kaplan-Meier curve for progression-free survival (PFS) of the 48 EOC patients stratified on MSLN expression according to the median cut-off, *p* value calculated by log rank test. **d** Boxplots showing a significantly higher *MSLN* expression in the serous subtypes compared with other subtypes of EOC in Tothill (*n* = 277), Denkert (*n* = 80) and Hendrix (*n* = 99) data sets, *p* values were calculated by Student’s *t* test. **e** Representative immunocytochemistry and immunofluorescence images for MSLN and p53 in primary and matched peritoneal metastasis of HGSC. Scale bar 100 µm and 20 µm for immunocytochemistry and immunofluorescence images, respectively. **f** MSLN immunocytochemistry results from primary and matched peritoneal metastasis of HGSC, in 19 cases from Centro Hospitalar de São João (CHSJ) and 5 cases from Portuguese Oncology Institute of Porto (IPOP). **g** Western blot data for PAX8 and MSLN expression in human cell lines: fallopian tube secretory epithelial cells (FT190 and FT194), ovarian cancer (OVCAR3, OVCAR4, OVCAR8, BG1, Kuramochi, A2780, TOV112D, TOV21G, EFO27, SKOV3, and IGROV1) and human ovarian surface epithelial cell(s)(HOSE6–3). **h** Graphical representation of *MSLN*-editing using two different sgRNAs (red and green) targeting the exon 2, deleting a genomic locus up and downstream of the translation start site including a part of the open reading frame (ORF) of *MSLN*. **i** Representative Western blot shows loss of MSLN expression in OVCAR8 Δ*MSLN* clones and re-expression of EGFP and MSLN in Δ*MSLN* OVCAR8 cells after rescue/overexpression (rescue). **j** Depiction of the construct used to establish cell lines with stable expression of EGFP-MSLN.

MSLN is overexpressed in ovarian^7,8^, pancreatic^9^ and gastric carcinomas^10^ and in mesotheliomas^17^. We first conducted a search on MSLN expression and clinical impact on cancer in general, and ovarian cancer in particular. Accessing The Cancer Genome Atlas (TCGA) database we found that cancer patients (of all cancer types) with high *MSLN* expression experienced poorer outcome with shorter overall survival (*n* = 8901, *p* < 0.001, hazard ratio 1.9 (CI 1.7–2.1) and relapse-free survival (*n* = 5664, *p* < 0.001, hazard ratio 1.6 (CI 1.4–1.8) (Fig. S1A, B, respectively). In addition, we found that ovarian cancer has the highest *MSLN* expression among the TCGA cancer types (Fig. S1C). Ovarian cancer comprises a heterogeneous group of malignant tumors, with epithelial ovarian cancer (EOC) being the most frequent histological type, accounting for approximately 90% of all cases^18^. EOC can be further divided into five major subtypes, that differ in respect to essential features including pathogenesis and prognosis^19^. Then, we studied the MSLN protein expression across the different subtypes of EOC and found a significantly higher expression in serous cancer subtypes (including high and low-grade serous carcinomas) (Fig. 1a). High MSLN expression was also associated with the presence of ascites at primary diagnosis (Fig. 1b), an indicator of peritoneal dissemination^20,21^, and shorter progression-free survival of EOC patients (Fig. 1c). Accessing three independent transcriptomic data sets^22–24^ confirmed that serous cancer subtypes (including high and low-grade carcinomas) have higher levels of *MSLN* than other subtypes of EOC (Fig. 1d). Since HGSC, is the most frequent and aggressive histological subtype of EOC^25,26^ and is frequently associated with peritoneal carcinomatosis, we studied MSLN protein expression in three independent series. Immunocytochemistry evaluation of 64 cases of HGSC showed that 70.3% of the cases had MSLN overexpression (*Allred* score 7 and 8) (Fig. S1D). In a subseries of 24 cases of HGSC we had access to matched peritoneal metastasis and observed that metastases share the MSLN overexpression levels of primary HGSC (Fig. 1e, f). These observations, showing high expression of MSLN sustained in the metastization process together with the impact on cancer behavior, increased our interest to dissect the role of MSLN in the peritoneal dissemination process. In order to setup an experimental model, we studied MSLN expression in eleven ovarian cancer cell lines, two human fallopian tube secretory epithelial cell lines and one human ovarian surface epithelial cell line (Fig. 1g). PAX8, a Müllerian lineage marker expressed by fallopian tube secretory epithelial cells that has been used for identification of the origin of serous ovarian and tubal cancers^27^, was incorporated in the screening to evaluate the phenotypic similarity between primary tumors and cell lines. Then, we selected three MSLN^high^ (OVCAR3, OVCAR8, and Kuramochi) and two MSLN^low^ (OVCAR4 and BG1) ovarian cancer cell lines to generate *MSLN* knockout *(*Δ*MSLN*) and constitutively MSLN overexpression (MSLN OE) cells, respectively. The CRISPR-*Cas9* gene editing technology was used to homozygously delete a genomic region that comprises the entire exon 2 and part of the translation start site of *MSLN* (Fig. 1h). We successfully established homozygous knockouts for *MSLN* in OVCAR3 and OVCAR8 cell lines using our paired sgRNA strategy (Fig. S2A, B and Supplementary Table S1). The presence of indels did not influence Δ*MSLN* confirmed by Western blot showing the entire loss of MSLN in the Δ*MSLN* clones (Figs. 1i, S2C). In order to reversibly alter possible MSLN-specific phenotypes, we lentivirally transduced either MSLN^low^ BG1 and OVCAR4 or Δ*MSLN* OVCAR8 ovarian cancer cells to overexpress or rescue the full length MSLN, respectively (Fig. 1i, j and S2D-F). The proper localization of MSLN protein in the rescue/overexpressing cell lines was confirmed by immunocytochemistry studies (Fig. S3).

### MSLN triggers anoikis resistance and anchorage-independent cell growth

**Fig. 2:**
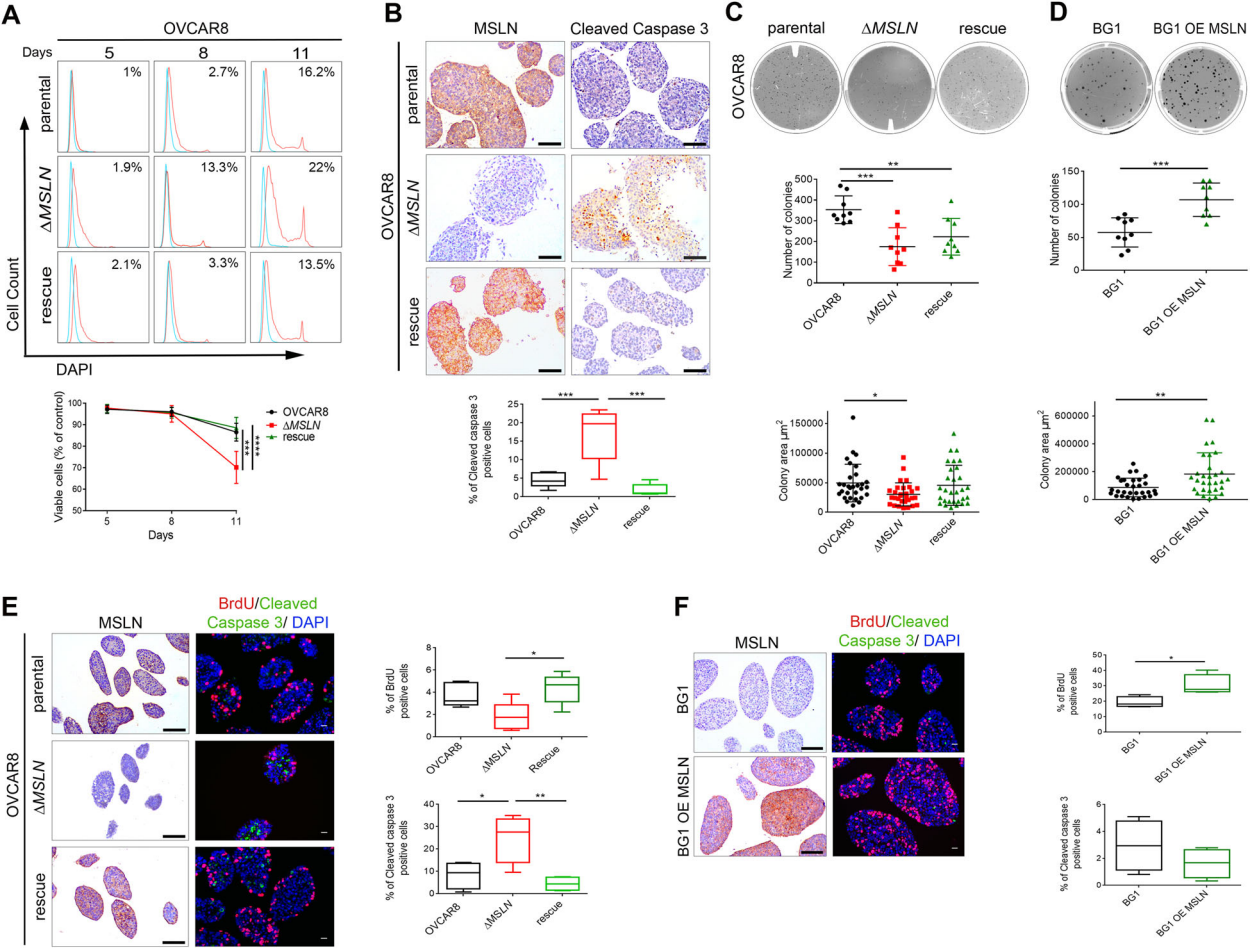
MSLN drives anoikis resistance and anchorage-independent growth. **a** Histogram for cell-detachment-induced apoptosis (anoikis) for OVCAR8 cells. Unstained or negative control (blue) and percentage of DAPI stained cells (dead cells, red). The percentage in each histogram refers to the DAPI positivity. Line chart showing the percentage of viable cells–DAPI negative cells, between day 5 and day 11. **b** Representative immunocytochemistry images for cleaved caspase 3 and MSLN of OVCAR8 cells cultured for 11 days in low adhesion conditions. Boxplots showing the percentage of cleaved caspase 3 positive cells. **c** and **d** Representative wells from anchorage-independent cell growth assay and dot blots showing the quantification of number (*n* = 9 wells) and area of cell colonies (*n* = 30 colonies) for OVCAR8 (**c**) and BG1 (**d**) cells. **e, f** Representative immunocytochemistry and immunofluorescence images for MSLN, BrdU and cleaved caspase 3 of OVCAR8 (**e**) and BG1 (**f**) cell colonies obtained from soft agar assays. Boxplots showing the quantification of BrdU and cleaved caspase 3 positive cells. Quantification of BrdU and cleaved caspase 3 positive cells was done using QuPath software. Dot blots and line charts are shown as mean ± SD and boxplots are shown as median and interquartile range of 3 (**a**, **c**, **d, e** and **f**) or 2 (**b**) independent experiments. *p* values were calculated by two-way ANOVA followed by Turkey’s multiple comparison test (**a**), one-way ANOVA followed by Turkey’s multiple comparison test (**b**, **c**, and **e**) and unpaired, two-tailed-*t* test (**d**, **f**) (*p < 0.05, **p < 0.01, ***p < 0.001, ****p < 0.0001). Scale bar 100 µm for immunocytochemistry images and 20 µm for immunofluorescence images. OE, overexpression.

Anoikis resistance is a vital trait for metastatic progression and is typical for ovarian cancer cells that survive in the ascitic fluid prior to metastatic colonization^28^. Thus, we evaluated the influence of MSLN on cell detachment-induced apoptosis (anoikis). We observed a significant decrease in cell viability for Δ*MSLN* compared with parental ovarian cancer cells after 11 days (OVCAR8) or 8 days (OVCAR3) of culture under low-adhesion conditions (Figs. 2a and S4A). Importantly, the anoikis sensitivity obtained upon *MSLN* deletion could be fully rescued in OVCAR8 cells (Fig. 2a). In accordance with the reduction on cell viability in the Δ*MSLN* cells, we observed an increase in the percentage of positive cells for cleaved caspase 3 indicating that cells loosing MSLN undergo increased apoptosis (Figs. 2b and S4B). The differences observed in anoikis resistance were further investigated in the anchorage-independent cell growth assay, using soft agar plates. The ability to exhibit anchorage-independent cell growth has been considered to be fundamental in cancer biology, linking the anoikis resistance with the metastatic capacity of cancer cells^29^. A significant reduction in the number and size of cell colonies from soft agar assays, was observed for Δ*MSLN* compared with parental OVCAR8 cells (Fig. 2c). The rescue of MSLN expression on Δ*MSLN* OVCAR8 partially restored the anchorage-independent cell growth capacity in those cells (Fig. 2c). Since parental and Δ*MSLN* OVCAR3 cells were unable to grow in this assay, we bicistronically overexpressed (OE) EGFP and MSLN in BG1 cells (MSLN^Low^) (Fig. S2D). A significant increase in the anchorage-independent cell growth (number and size of cell colonies) was observed for BG1 OE MSLN as compared with control cells (Fig. 2d). Together with the decrease in colony size, we also observed a significant reduction in the number of proliferative cells in the context of reduced MSLN expression (Fig. 2e, f). To evaluate the effect of MSLN on cell proliferation under 2D culture conditions, we performed a cell proliferation/viability assay in standard monolayer cultures. Here, we observed that MSLN expression significantly increases proliferation/viability of OVCAR3 and BG1 cells but had no effect on OVCAR8 cells (Fig. S4C-E). Taken together, our results indicate that MSLN expression promotes not only cell survival but also cell proliferation under anchorage-independent conditions.

### MSLN enhances single and aggregate in vitro cell invasion and co-culture-based mesothelial clearance

**Fig. 3:**
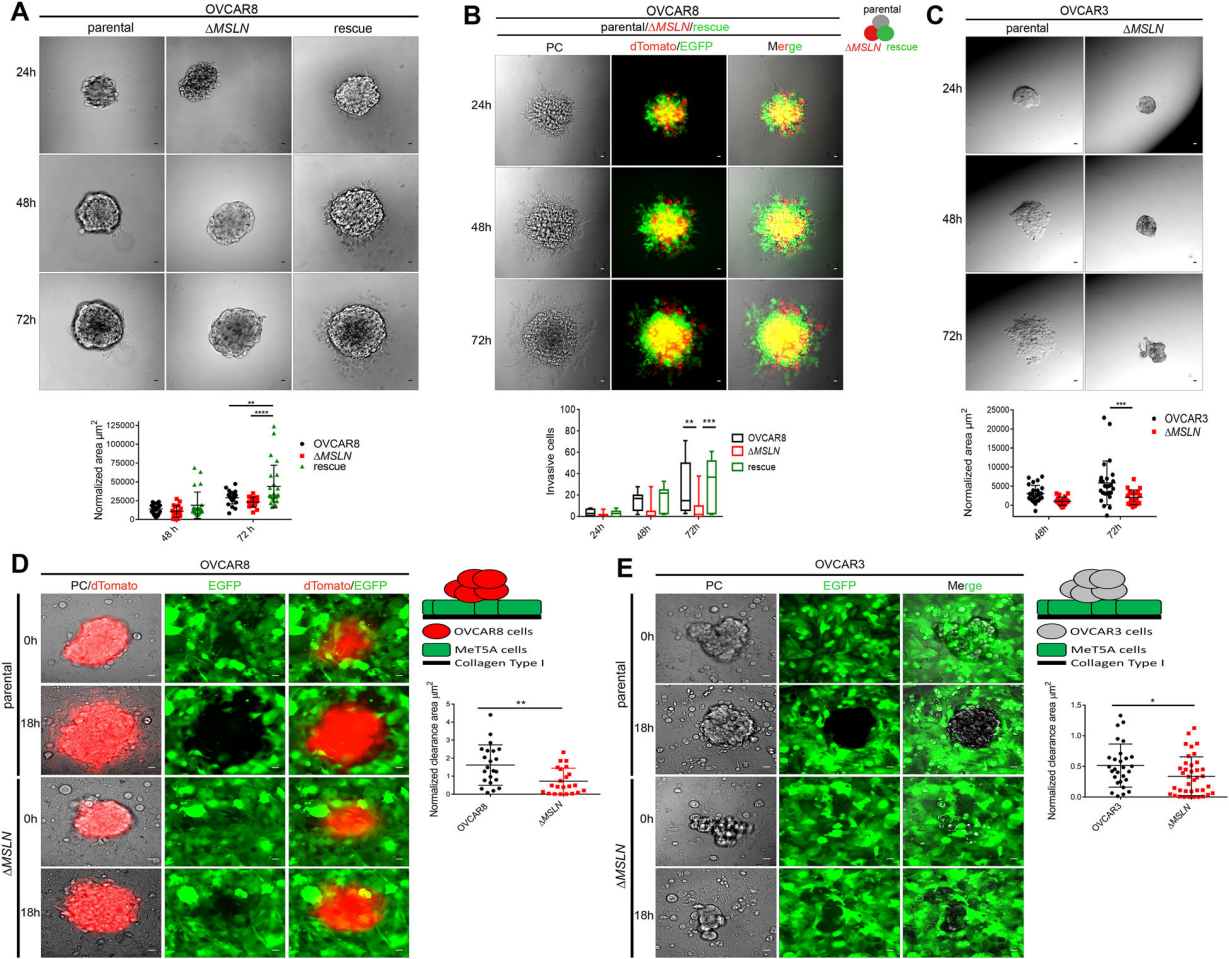
MSLN enhances cancer cell invasion and mesothelial clearance. **a** Representative phase-contrast images of aggregate cell invasion assay for OVCAR8 cells. Dot plot showing the quantification of invasive area after 48 and 72 h of incubation. The invasive area was normalized for the aggregate size at 24 h of incubation. **b** Representative phase-contrast and fluorescence images for competition aggregate cell invasion assay. Equal amounts of the three cell populations, parental, Δ*MSLN* (expressing dTomato) and Δ*MSLN* rescue/overexpression (expressing EGFP) OVCAR8 cells, were used for the aggregate formation. Boxplots showing the quantification of the number of invasive cells for parental, Δ*MSLN* (expressing dTomato) and Δ*MSLN* rescue/overexpression (expressing EGFP) OVCAR8 cells at 24, 48. and 72 h of incubation. **c** Representative phase-contrast images of aggregate cell invasion assay for OVCAR3 cells. Dot plot showing the quantification of invasive area after 48 and 72 h of incubation. **d, e** Representative images of mesothelial clearance assay for OVCAR8 (**d**) and OVCAR3 (**e**) cell aggregates. Dot blots showing the normalized clearance area of OVCAR8 (**d**) and OVCAR3 (**e**) cell aggregates. After 18 h of co-culture, the negative space created in the mesothelial monolayer by the ovarian cancer cell aggregates was measured and divided by the initial size of the ovarian cancer cell aggregates at time 0 to determine the normalized clearance area. Data are shown as mean ± SD of 3 (**a**, **c**, **d**, **e**) and 2 (**b**) independent experiments; more than 7 aggregates were used per condition/experiment. *p* values were calculated by two-way ANOVA followed by Turkey’s multiple comparison test (**a** and **b**) or Sidak’s multiple comparison test (**c**) and unpaired, two-tailed-*t t* test (**d**, **e**) (*p < 0.05, **p < 0.01, ***p < 0.001, ****p < 0.0001). Scale bar 20 µm. PC phase-contrast.

Ovarian cancer is characterized by early dissemination/invasion of cancer cells to the organs located in the peritoneal cavity^28,30^. To determine the role of MSLN in ovarian cancer cell invasion we used single and aggregate cell invasion assays. In the 2D transwell assays, we observed a significant decrease in the number of single invasive cells for Δ*MSLN* compared with parental cells (Fig. S5A, B). Of note, the rescue of MSLN expression on Δ*MSLN* partially restored the 2D invasion capacity of OVCAR8 cells (Fig. S5A). Cell clusters have been described as the predominant organization of metastatic cells in the peritoneal cavity^31^. To mimic this process, we generated aggregates of our cell lines to test their invasion capacity on Matrigel^®^. Here, we observed that cell invasion varies according to MSLN expression, with more invasion in Δ*MSLN* rescue/overexpression followed by parental and lastly the Δ*MSLN* OVCAR8 cells (Fig. 3a). The reduced invasion ability of Δ*MSLN* OVCAR8 cells was confirmed in a competition aggregate cell invasion assay where we use a mixture of equal cell numbers of the three OVCAR8 cell populations (parental without fluorescent protein, Δ*MSLN* with dTomato, Δ*MSLN* rescue/overexpression with EGFP) for the aggregate formation (Fig. 3b). Using the chorioallantoic membrane (CAM) invasion in vivo assays in OVCAR8 cells, we further confirmed the reduction in the invasive capacity of Δ*MSLN* as compared with parental OVCAR8 cells (Fig. S6). Similar results were also obtained for OVCAR3 with decreased aggregate cell invasion capacity in Δ*MSLN* OVCAR3 cells (Fig. 3c). However, the smaller invasive area observed in Δ*MSLN* OVCAR3 cells may be related to impaired proliferation of OVCAR3 cells upon MSLN knockout. The formation of peritoneal metastasis depends on the ability of cell clusters to adhere to distant organs in the peritoneal cavity. This process entails initial adhesion followed by invasion through the mesothelial cell monolayer^32^. Mesothelial clearance assays have been used as an in vitro co-culture model to evaluate the ability of ovarian cancer cell aggregates to attach and invade the mesothelial monolayer^33–35^. We then co-cultured aggregates of parental and Δ*MSLN* ovarian cancer cells with monolayers of mesothelial cells stably expressing EGFP, MeT5A-EGFP. Of note, the mesothelial clearance assays were performed in a time-window in which no significant differences in cell proliferation/anoikis resistance was observed between parental and Δ*MSLN* cells (Fig. 2a and Fig. S4A, C and D). As shown in Fig. 3d, e, a significant decrease in mesothelial clearance capacity was observed in Δ*MSLN* clones as compared with parental cells. The role of MSLN on the mesothelial clearance was further evaluated using parental and rescue OVCAR8 cells, where we observed that rescuing the MSLN expression restores the mesothelial clearance capacity of OVCAR8 cells (Fig. S7). These data indicate that MSLN regulates the ability of ovarian cancer cells to adhere, breach and invade the mesothelium.

### MSLN triggers increased tumor burden and peritoneal dissemination in nude mice

**Fig. 4:**
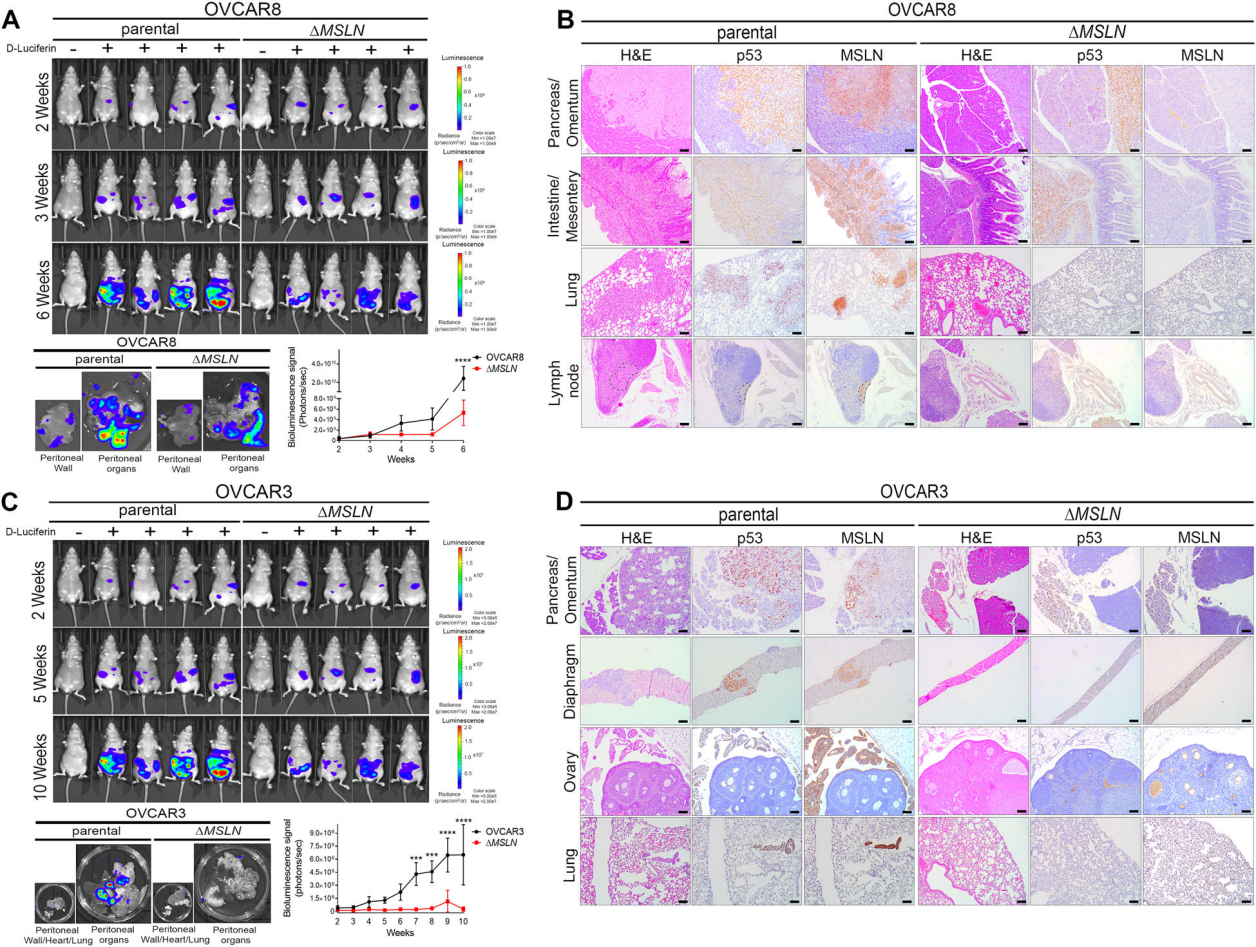
MSLN enhances tumor burden and peritoneal dissemination. **a**, **c** Representative bioluminescence images for OVCAR8 (*n* = 8 per group) (**a**) and OVCAR3 (*n* = 6 per group) (**c**) xenografts. Bioluminescence signals in the peritoneal organs, peritoneal wall (**a** and **c**), lung/heart (**c**) are also shown. Line chart showing the quantification of bioluminescence signals (photons/s) between week 2 and week 6 (**a**) or week 10 (**c**) after intraperitoneal injection of OVCAR8 and OVCAR3 luciferase labeled cells. Bioluminescence signals were measured continuously at 1 min intervals 5 min after subcutaneous injection of 100 µl of D-Luciferin at 20 mg/ml. Data are shown as mean ± SD. *p* values were calculated by two-way ANOVA followed by Sidak’s multiple comparison test (**a**, **c**) (***p < 0.001, ****p < 0.0001). **b, d**, Representative metastatic sites shown by H&E staining for OVCAR8 and OVCAR3 xenografts. Immunocytochemistry of p53 was used to better identify cancer cells and MSLN staining to confirm the absence of MSLN expression in Δ*MSLN* from OVCAR8 (**b**) and OVCAR3 (**d**) cells. Scale bar 100 µm.

The results obtained from our in vitro studies suggest that MSLN contributes to the metastatic potential of ovarian cancer cells by regulating anoikis resistance, anchorage-independent cell growth, invasion and mesothelial clearance. In order to translate our in vitro findings in an in vivo model, we assessed to what extent MSLN drives tumor growth and peritoneal dissemination in nude mice. Parental and Δ*MSLN* ovarian cancer cells were reengineered to stably express luciferase, allowing monitoring of tumor burden and dissemination over time (Fig. S8A, B). Parental and Δ*MSLN* luciferase labeled cells were injected intraperitoneally into NIH(S) II: *nu/nu* mice. Six weeks after injection, we observed significantly lower levels of bioluminescence signals for Δ*MSLN* compared with parental OVCAR8 xenografts (Fig. 4a). Histological examination of peritoneal organs, despite showing a similar number of animals with implants for parental/Δ*MSLN* OVCAR8 xenografts (Fig. S8C), revealed a reduction in invasive capacity of Δ*MSLN* cells in pancreas/omentum and intestine/mesentery (Fig. 4b). Lymphatic metastases were detected in thoracic (1/8) and intraperitoneal (2/8) lymph nodes and haematogeneous metastases were detected, as multiple metastatic foci, in the lung of 1/8 parental OVCAR8 xenografts (Fig. 4b, Fig. S8C). Similarly, when compared to parental, Δ*MSLN* OVCAR3 xenografts showed significantly lower bioluminescence signals after seven weeks of intraperitoneal injection (Fig. 4c). Histological evaluation of the peritoneal organs revealed a striking decrease in tumor implantation and invasion for Δ*MSLN* compared with parental OVCAR3 xenografts (Figs. 4d and S8D). Haematogeneous metastases were identified as multiple metastatic foci, in the lung of 2/6 parental OVCAR3 xenografts (Figs. 4d and S8D). We also established intraperitoneal xenografts for BG1 and OVCAR4 cell lines (both are MSLN^low^) with and without MSLN overexpression and observed again that tumor burden and dissemination was enhanced through MSLN overexpression (Fig. S9). In addition, to evaluated if differences in cell proliferation between parental and Δ*MSLN* or overexpressing cells could be related to the difference observed in tumor burden and dissemination, we studied the number of Ki67 positive cells in peritoneal implants from the xenografts and found no significant differences (Fig. S10). To reinforce the role of MSLN in the peritoneal dissemination process, we compared the peritoneal metastatic potential in nude mice of ovarian cancer cell lines that “naturally” express high levels of MSLN with ovarian cancer cell lines that “naturally” express low levels of MSLN. Overall, we observed that MSLN^high^ cell lines (OVCAR8, OVCAR3, and IGROV1) have an increased peritoneal dissemination capacity as compared to MSLN^low^ (BG1, OVCAR4, and SKOV3) cell lines (Fig. S11). Collectively, these results indicate that MSLN expression triggers increased tumor burden and dissemination of ovarian cancer cells in vivo.

## Discussion

**Fig. 5:**
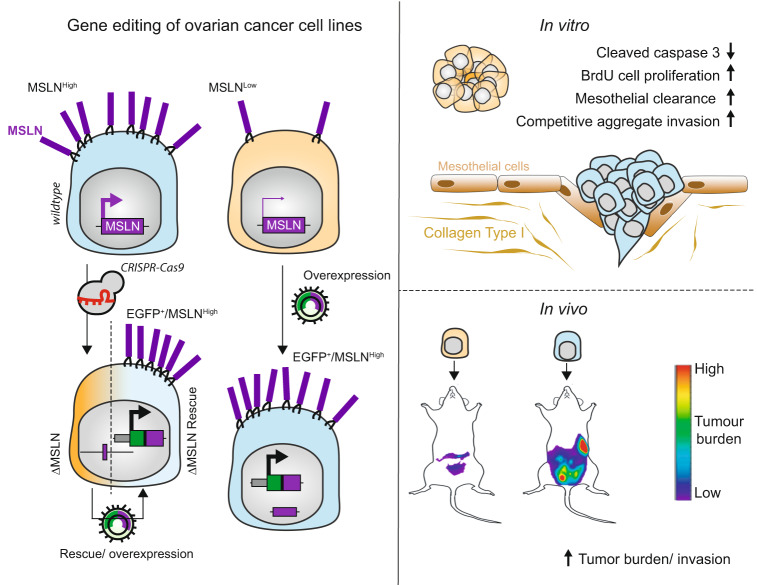
Summarized working model of MSLN-promoted ovarian cancer metastasis. Loss of MSLN upon homozygous genomic deletion reversibly alters cancer cell survival and growth in suspension, invasion of cell clusters through the mesothelial layer and spreading to organs within the peritoneal cavity.

Metastization to the peritoneal cavity is frequently observed in ovarian cancer and is a major cause for the unfavorable outcome and poor prognosis in patients with HGSC^6,28^. Our aim was to address the essentially unknown role of MSLN in the intraperitoneal dissemination of ovarian cancer. In this study, we provide evidence indicating that MSLN is a key player in peritoneal dissemination of ovarian cancer by promoting cell survival in suspension, mediating invasion of cell clusters through the mesothelial layer and spreading to organs within the peritoneal cavity (Fig. 5).

Pre-clinical and clinical studies increasingly show that aberrant MSLN expression plays a role in cancer progression^13,15,36,37^. In the present study, we found that high MSLN expression levels are associated with the presence of ascites at the time of the diagnosis in EOC patients. Importantly, the presence of ascites has been correlated with the peritoneal spread of ovarian cancer, the major cause of unfavorable outcome and poor prognosis of EOC patients^20,21^. High MSLN expression in HGSC has been reported in previous studies^38,39^ and here we show for the first time that almost all HGSC have elevated levels of MSLN both at the primary and matched metastatic sites. These observations indicate that MSLN overexpression, in the setting of HGSC, describes a stable phenotype during cancer progression, which is in line with its possible role in the dissemination process.

Using genetically engineered cell line models, we demonstrated that MSLN expression promotes not only anoikis resistance but also increased proliferation under anchorage-independent conditions. Anoikis resistance is typically observed in cancer cells that survive in the ascitic fluid prior to metastatic colonization^28^. Moreover, the ability to exhibit anchorage-independent cell growth links anoikis resistance to the metastatic capacity of cancer cells^29^. The role of MSLN in anoikis resistance that we observed is not restricted to ovarian cancer cells. In fact, other studies using breast and lung cancer cell lines have demonstrated that MSLN expression confers an advantage to cell survival and proliferation in suspension^40,41^, which is a feature that may be important for the peritoneal dissemination of floating cancer cells in the peritoneal cavity.

Our study provides evidence that MSLN triggers cell invasion ability. In vitro cell invasion has been typically evaluated in transwell assays using standard cultures. However, in ovarian cancer, cell aggregates are critical for effective metastatic dissemination within the peritoneal cavity^31^. To mimic this process, we used an aggregate cell invasion assay and have shown that MSLN levels regulate the invasive capacity of ovarian cancer cells. Successful implantation also depends on the ability of cancer cell aggregates to adhere and invade the mesothelial cell monolayer^42^. The use of the co-culture mesothelial clearance assay demonstrated that MSLN enhances the ability of cancer cell aggregates to adhere, breach, and invade the mesothelial cell layer. In addition to the role of MSLN in the mesothelial clearance process that we detected, other mechanisms also contribute to successful mesothelial clearance. Iwanicki et al. reported that ovarian cancer cells use the axis α5β1 integrin, myosin II, and talin I to achieve mesothelial clearance^34^. The same group identified a gene signature enriched in mesenchymal genes on mesothelial clearance-competent cell aggregates^43^. Recently, SUSD2^44^, ADH1B^45^ proteins and *O*-linked glycosylation^33^ have also been identified as players in the mesothelial clearance process.

Our in vitro findings jointly indicate that MSLN contributes to the metastatic potential of ovarian cancer cells by regulating anoikis resistance, anchorage independent cell growth, invasion and mesothelial clearance. To further explore the role of MSLN in the metastatic potential of ovarian cancer cells in vivo, we developed a model using luciferase labeled ovarian cancer cells and IVIS Lumina in vivo imaging as a non-invasive approach to track tumor burden and dissemination over time. Consistent with the in vitro results, we demonstrated that elevated levels of MSLN enhances tumor burden, invasion and dissemination of ovarian cancer cells when intraperitoneally injected into nude mice. These results fit with previous clinical observations describing an association between MSLN overexpression and ovarian^15^, pancreatic^46^ and gastric^10^ tumor aggressiveness. In gastric cancer, it was also reported that MSLN expression led to increased lymph node metastasis and blood vessel invasion^10^, which is in line with the results from the present study where haematogenous/lymphatic metastasis were mainly detected in the xenografts established with MSLN^high^ ovarian cancer cell lines. Interestingly, very recently Avula and collaborators reported that MSLN enhances the tumor vascularity of newly formed pancreatic peritoneal metastases^47^. Collectively, these findings suggest that further than promoting cell survival in suspension, invasion through the mesothelial layer and spread within the peritoneal cavity, MSLN may play a role in other processes such as angiogenesis and haematogenous/lymphatic dissemination.

In ovarian and pancreatic cancers, the role of MSLN in tumor progression/invasion has been associated to MUC16 (CA125), the only described binding partner of MSLN. A MSLN-MUC16 based homophilic and heterophilic cell-cell interaction has been reported to be important for the contact between cancer cells, as well as cancer and mesothelial cells. Moreover, MUC16-MSLN interaction can also trigger tumor invasion through MMP-7 activation^37,48^. We observed a pro-tumorigenic role of MSLN, in MUC16 deficient (OVCAR8) and proficient (OVCAR3, BG1, and OVCAR4) ovarian cancer cell lines^33^ which suggests that MSLN can act in a MUC16-independent manner. In fact, MSLN overexpression alone has been reported to be associated with activation of NFkB, MAPK, PI3K, and ERK signaling pathways promoting cell proliferation, anoikis resistance and invasion^13,37,49^. In line we the results from Cheng et al.^13^, we observed an increase in the activation (phosphorylation) of ERK1/2 and induction of MMP-7 expression in response to elevated levels of MSLN in OVCAR3 and BG1 cell lines models. However, we did not observe a similar effect in OVCAR8 and OVCAR4 cell lines (Fig. S12). These results suggest that the mechanism of MSLN action involves multiple signaling pathways dependent on the cell line. Thus, additional studies are needed to fully elucidate the signaling pathways that are under control of MSLN.

Finally, MSLN is emerging as an attractive target for cancer immunotherapy, considering its overexpression in several tumor contexts^50,51^. Several immunotherapeutic strategies have been devised for MSLN, including antibody-based therapies and adoptive T-cell therapy (CAR T cells)^50,52–55^ with some promising results. Our study provides additional support for considering MSLN targeted therapies in HGSC, since, as we show here, it is a strong element that promotes tumor aggressiveness and more than 70% of HGSC and peritoneal metastasis displayed MSLN overexpression. However, heterogeneous intratumoural MSLN expression (up to 25% of tumor cells without MSLN expression, data not shown) was observed in this study, and our group is trying to identify compounds that are more efficient in MSLN negative/positive ovarian cancer cells, that might be valuable to use in combination with MSLN targeted therapies.

Collectively, our data demonstrate that MSLN contributes to the uniqueness of ovarian cancer dissemination in the peritoneum by promoting cell survival in suspension, invasion through the mesothelial cell layer and spreading within the peritoneal cavity, providing support for further clinical investigation of MSLN as a therapeutic target in HGSC.

## Material and methods

### Patients and specimens

A retrospective series of 20 HGSC, diagnosed between 2002 and 2015, was retrieved from the archives of the Pathology Department of Centro Hospitalar de São João (CHSJ series). Cases were selected based on the quality/representability of the histological material, clinical information and histological type (WHO classification). In 19 cases from this series, paraffin blocks of peritoneal metastasis were also available.

Eight HGSC from the Pathology Department of the Portuguese Oncology Institute of Porto (IPOP series) diagnosed between 2011 and 2017, were selected form an original retrospective series composed by EOC with cytological material from ascitic fluid embedded in paraffin blocks. In 5/8 HGSC, paraffin blocks of peritoneal metastasis were also available.

Forty-eight EOC were available for study in the Ovarian cancer research group, University Hospital of Basel and University of Basel (Basel series).

### Cell lines

Immortalized human fallopian tube secretory epithelial cell lines (FT190 and FT194) were cultured in DMEM-Ham’s F12 (Sigma-Aldrich) without HEPES Buffer, 1% penicillin/streptomycin (Sigma-Aldrich) and 2% Ultroser^Tm^ (PALL, Life Science). Immortalized human ovarian surface epithelial cell line (HOSE6–3) and ovarian cancer cell lines (OVCAR3, OVCAR4, OVCAR8, BG1, Kuramochi, A2780, TOV112D, TOV21G, EFO27, SKOV3, and IGROV1) were cultured in RPMI 1640 (Thermo Fisher Scientific) containing 10% fetal bovine serum (FBS) (Biowest) and 1% of penicillin/streptomycin (Thermo Fisher Scientific). Immortalized human mesothelial cell line MeT5A (ATCC, American Type Culture Collection) was cultured as previously described^33^. All cell lines were maintained at 37 °C and 5% CO_2_. In prior experiments, all cell lines were authenticated using short tandem repeat (STR) profiling (PowerPlex 16 HS kit, Promega) and regularly tested for the absence of mycoplasma.

### Immunocytochemistry and immunofluorescence

Immunocytochemistry and immunofluorescence studies were performed as previously described^33^. A detailed description is provided in Supplementary Information. The list of antibodies used is provided in Supplementary Table S2.

### CRISPR-*Cas9* mediated *MSLN* knockout and MSLN rescue/overexpression

A detailed description of the CRISPR-*Cas9* design, molecular cloning, cell sorting strategy, characterization of homozygously deleted cancer cells, and MSLN rescue/overexpression is provided in Supplementary Information.

### In vitro assays for characterization of *MSLN* knockout and MSLN rescue/overexpression cells

A detailed description of in vitro assays for studying cell proliferation, anchorage independent cell growth, anoikis resistance, in vitro and in vivo cell invasion, mesothelial clearance, protein extraction and western blot analysis is provided in Supplementary Information.

### Establishment of intraperitoneal xenograft models in nude mice

Prior to injection, bioluminescence signals from the ovarian cancer cell lines, transduced with pUltra-Chili_Luc plasmid, were evaluated using the IVIS Lumina in vivo imaging system (Perkin Elmer) (Fig. S8B and S9A). To generate intraperitoneal xenografts 10 × 10^6^ of OVCAR8 cells, 14 × 10^6^ of OVCAR3 cells, 6 × 10^6^ of BG1 cells, 8 × 10^6^ of OVCAR4 cells, 2 × 10^6^ of IGROV1 cells and 4 × 10^6^ of SKOV3 cells in 200 µl of PBS were intraperitoneally injected, using 25 gauge needles, in female NIH(S) II: *nu/nu* mice with 6–8 weeks of age. In the experiments using parental and ∆*MSLN* or OE MSLN cells, mice were randomly divided into two groups. Bioluminescence signals were evaluated, in blind fashion manner, continuously at 1 min intervals, 5 min after subcutaneous injection of 100 µl of D-Luciferin (PerkinElmer) at 20 mg/ml once a week. At the end of the experimental period, mice were humanely euthanized and peritoneal and pleural cavities were carefully inspected. Animal organs were harvested for histological processing. H&E staining from all tissue blocks were examined under the optical microscope to evaluate tumor localization, growth and invasion. All immunostaining procedures in mice tissues were performed according to the same protocols described above.

### Statistics

Statistical analyses of the transcriptomic data sets were performed using RStudio software version 1.2.1335 (https://www.rstudio.com). Kaplan-Meier method with log-rank test was used for comparison of survival curves in all TCGA cancer types. The threshold for overall and relapse-free survival was determined by using conditional inference tree model (R package ‘party’). To ensure adequate power to detect a pre-specified effect, all sample sizes were chosen based on initial pilot experiments, including the main animal studies. No samples or animals were excluded from the analysis. Statistical analyses of the in vitro and in vivo experiments were performed using the GraphPad Prism v6 (GraphPad Holdings, LLC, USA). A 2-tailed Student’s *t* test was used for comparison between 2 groups, one-way and two-way ANOVA with Sidak’s or Turkey’s multiple comparisons test were used for multi-group comparisons. The level of statistical significance was set at a *p* value of less than 0.05. Data were reported as mean ± SD or as median and interquartile range.

### Study approval

All human samples, were selected in accordance with the local ethical guidelines (as stipulated by the Declaration of Helsinki) being approved by the Ethical Committee from Centro Hospitalar de São João (CHSJ) (Ref.86/2017), Portuguese Oncology Institute of Porto (IPOP) (Ref.92R1019) and by the Swiss Medical Ethical Committee (EKNZ 2015–436).

All procedures in animals were performed in accordance with the European Guidelines for the Care and Use of Laboratory Animals, Directive 2010/63/UE, Portuguese National Regulation (Decreto-Lei n.8 113/ 2013 de 7 de Agosto) and approved by the local Ethics Committee of the Institute for Research and Innovation in Health (i3S). Project identification code 0421/000/000/2017, date (24/05/17). NIH(S)II: *nu/nu* mice, strain described by Azar, H.A et al.^56^, were generated under IPATIMUP supervision.

## Supplementary information

Supplementary Information

## Data Availability

TCGA data sets were accessed through the UCSC Cancer Genomics Browser website as recently described^57^. Additional publicly available transcriptomic data sets were downloaded from Gene Expression Omnibus (http://www.ncbi.nlm.nih.gov/geo/). Data sets were analyzed and figures were obtained through RStudio software version 1.2.1335 (https://www.rstudio.com). The following transcriptomic data sets for analysis of *MSLN* expression among different histotypes Hendrix (GSE6008, histotypes: *n*_others_ = 58, *n*_serous_ = 41)^24^, Tothill (GSE9899, histotypes: *n*_others_ = 21, *n*_serous_ = 256)^22^, Denkert (GSE14764, histotypes: *n*_others_ = 12, *n*_serous_ = 68)^23^.
