# Supplementary material for: Regulation of invasion and peritoneal dissemination of ovarian cancer by mesothelin manipulation
Source: Oncogenesis. 2020 Jul 1;9(6):61. doi: 10.1038/s41389-020-00246-2 (PMC7329842; doi:10.1038/s41389-020-00246-2)
Supplement: Supplementary file 1 — Supplementary Information [file 41389_2020_246_MOESM1_ESM.docx]

**Regulation of invasion and peritoneal dissemination of ovarian cancer by mesothelin manipulation**

Ricardo Coelho,^1,2,3^ Sara Ricardo,^1,2,3^ Ana Luísa Amaral,^1,2^ Yen-Lin Huang,^4^ Mariana Nunes,^1,2,5^ José Pedro Neves,^1,2,6^ Nuno Mendes,^2,7^ Mónica Nuñez López,^4^ Carla Bartosch,^8^ Verónica Ferreira,^8^ Raquel Portugal,^6^ José Manuel Lopes,^2,3,6,9^ Raquel Almeida,^1,2,3,10^ Viola Heinzelmann-Schwarz,^11^ Francis Jacob,^4^**^#^** Leonor David^1,2,3^ **^#*^**

**Supplementary information**

**Supplementary material and methods**

**Immunocytochemistry and immunofluorescence**

Formalin-fixed, paraffin-embedded tumour samples and cytoblocks from cell lines were sectioned with a standard microtome at 3- to 4-µm thickness. After deparaffinization, heat-induced (98°C) antigen retrieval was performed with citrate buffer (pH 6.0) (Thermo Fisher Scientific), and slides were incubated with hydrogen peroxide 3% (v/v). Next, slides were incubated with primary antibodies for 1 hour at room temperature (Supplementary Table S2). Primary antibodies were detected using a secondary antibody with horseradish peroxidase (HRP) polymer (Dako REAL™ EnVision™ Detection System). Visualization of the reaction was performed using diaminobenzidine according to the manufacturer's instructions and nuclei were counterstained with hematoxylin. For immunofluorescence, Alexa Fluor^TM^ 488 goat anti-Rabbit (Thermo Fisher Scientific) and Alexa Fluor^TM^ 594 goat anti-Mouse (Thermo Fisher Scientific) were used as secondary antibodies and nuclei were counterstained with DAPI**.** Immunocytochemistry and immunofluorescence for cell line samples was performed as previously described (1). Samples were then incubated with the primary antibodies, anti-BrdU to evaluate proliferative cells, anti-cleaved caspase 3 to evaluate apoptotic cells, and anti-MSLN for 1 hour at room temperature. Primary antibodies were detected using the secondary antibody with HRP polymer (Dako REAL™ EnVision™ Detection System) followed by incubation with diaminobenzidine according to the manufacturer's instructions and nuclei were counterstained with hematoxylin. For immunofluorescence, the same procedure as described above for tissue specimens was used**.** Immunofluorescence samples were examined in an inverted fluorescence microscope (Zeiss Axio Imager Z1), and images were acquired using Axiovision software (Carl Zeiss Inc.) at 200× magnification. Quantification of cleaved caspase 3 and BrdU positive cells was done using QuPath (v0.1.2) software (2).

**Immunocytochemistry Evaluation**

Immunocytochemistry slides were evaluated by two independent observers (LD and SR). MSLN membrane staining was scored by percentage of positive tumour cells and staining intensity, according to Allred *et al. (3)* scores 7 and 8 were considered to have MSLN overexpression.

**CRISPR-*Cas9* mediated *MSLN* knockout**

The design of single guide RNAs (sgRNAs) targeting the exon 2 of *MSLN* were carried out using the online program available from Zhang’s laboratory (4). SgRNA1 and sgRNA2 (Fig. 1h and Supplemental Table S1) with scores of 86 and 94 respectively, were selected for *MSLN* editing. Annealed sgRNAs were cloned into pSpCas9-2A-GFP (Addegene PX458) via *BsbI* restriction site using T4 DNA Ligase (Promega). Constructs were transformed into *DH5α* *E. coli* strain and sequenced for confirmation of the sgRNA insertion into PX458 by Sanger DNA sequencing using the primer human U6 (Supplemental Table S1). OVCAR8 and OVCAR3 cells were transiently transfected using Viafect transfection reagent (Promega) with 2.5µg of PX458 containing the specific sgRNA sequence. Cells were washed with PBS, harvested using a non-enzymatic cell dissociation buffer (Sigma-Aldrich) and finally resuspended in RPMI supplemented with 10% FBS, 72 hours after transfection. OVCAR8 cells were subjected to a single cell sorting for GFP^+^ cells into 96-well flat bottom plates using the BD FACS Aria Cell Sorter (BD Bioscience). For OVCAR3 cells, enrichment of GFP^+^ cells were performed 72 hours after transfection followed by a manually seeding as single cells into 96-well flat bottom plates. Plates were incubated for 3 to 4 weeks following transfer to 48-well plates and genomic DNA isolation for genotyping PCR to characterize single cell clones. Homozygous ∆*MSLN* clones were identified by two different PCRs using the appropriate primer pairs (Fig. S2A and Supplemental Table S1). PCRs were performed using 2x GoTaq green Master Mix (Promega), 200nM of each primer and 30ng genomic DNA. PCR conditions for the PCR_1 were 95°C for 2 minutes, then 32 cycles of 95°C for 30 seconds, 60°C for 30 seconds, 72°C for 1 minute, finishing with 1 cycle at 72°C for 10 minutes. PCR conditions for PCR_2 were 95°C for 2 minutes, then 32 cycles of 95°C for 15 seconds, 60°C for 15 seconds, 72°C for 15 seconds, finishing with 1 cycle at 72°C for 10 minutes. Amplicons were visualized on a 1-1.5% agarose gel. PCR products from the PCR_1 were purified and cloned into the pGEM^®^ T Easy Vector System (Promega) according to the manufacture’s protocol and sequenced using T7-F and SP6-R primers (Supplemental Table S1) to detect insertions and deletions at the *Cas9-*active sites (Fig. S2B).

**MSLN rescue/overexpression**

To rescue/overexpress MSLN, we used the pCMV3-MSLN (Sino Biological) plasmid as DNA template and designed primers to amplify the *MSLN* open read frame. The primers were designed to include an overhang sequence of *NheI* restriction site (Supplemental Table S1) for cloning into the pUltra-EGFP (Addgene #41393) backbone. The PCR was performed using 2U Platinum Taq DNA polymerase (Thermo Fisher Scientific), 1X Platinum Taq buffer, 400nM of forward and reverse primers, 200µM dNTPs, 100ng of plasmid DNA (pCMV3-MSLN) and nuclease free water under the following conditions: 95°C for 4 minutes followed by 32 cycles of 94°C for 45 seconds, 54°C for 45 seconds, 72°C for 3 minutes, finishing with 1 cycle at 72°C for 10 minutes. Amplicons were visualized on 0.8% agarose gel and purified by Wizard SV gel and PCR Clean/up System (Promega). The *MSLN* open read frame was then introduced into pUltra-EGFP via *NheI* cloning procedure followed by transformation into *Stbl3* *E. coli* strain. The right orientation and sequence of *MSLN* open read frame in the pUltra backbone (pUltra-EGFP-MSLN) was confirmed by Sanger DNA sequence (Microsynth) using the EGFP-C and WPRE-R primers (Supplemental Table S1).

**Lentivirus production and transduction**

For the lentivirus production we used the HEK293T cells, that were cultured in RPMI with 10% FBS and 1% penicillin/streptomycin. One day prior to transfection 4x10^6^ HEK293T cells were seeded into 75cm^2^ tissue culture flask. For each flask, 4µg of the plasmid encoding the gene of interest pUltra-EGFP-MSLN, pUltra-EGFP, pUltra-Chili (Addgene #48687), pUltra-Chili-Luc (Addgene #48688) and 2 µg of packaging (pCMVR8.74 Addgene #22036) and envelop (pMD2.G Addgene #12259) plasmids were transfected using 24µl of jetPEI reagent and 1ml of 150mM NaCl solution (Polyplus-transfection). Culture media was changed 24 hours after transfection. Supernatant containing active lentivirus was collected 48 hours later, filtered with a 0.45µm filter (Millipore), and stored at -80°C. Target cell lines were then transduced with the desired lentiviral supernatant in 3ml of media on a 25cm^2^ tissue culture flask. After 3 passages cells were sorted for EGFP/dTomato positive cells using the BD FACS Aria Cell Sorter (BD Bioscience).

**Single cell invasion assay**

5x10^4^ cells were seeded in the upper compartment of the transwell Matrigel-coated chambers with 8-µm pore-size membranes (BD Biosciences) in 500µl of RPMI supplemented with 1% FBS. In the lower compartment 750µl of RPMI containing 10% FBS was added as a chemoattractant, and incubated for 24 hours. Following incubation, non-invasive cells were removed with a cotton swab, whereas invasive cells were fixed in ice-cold methanol for 10 minutes. The membranes were carefully cut and mounted on microscope slides with Vectashield (Vector) with DAPI. The number of invasive cells was counted using an inverted fluorescence microscope (Zeiss Axio Imager Z1), and images were acquired using Axiovision software (Carl Zeiss Inc.) at 200× magnification.

**Aggregate cell invasion assay**

The aggregate cell invasion assay was preformed based on previous described protocols (5-7). For the aggregate formation, cells were seeded at the density of 300 cell/well into 96-well U bottom poly-HEMA coated plates and incubated for 24 hours. Following incubation, aggregates of ovarian cancer cells were collected, pelleted, resuspended in 50% of Matrigel^®^ and seeded into 8-well chambers (Ibidi) coated with 70µl of Matrigel^®^. Competition aggregate cell invasion assay was performed using the same conditions as aggregate cell invasion assay, except for aggregate formation. For the aggregate formation, we used a mixture of equal amounts (100 cells) of parental, ∆*MSLN* (expressing dTomato) and ∆*MSLN* rescue/overexpression (expressing EGFP) OVCAR8 cells. Aggregates were first incubated for 24 hours in the standard cell incubator followed by live cell imaging for 48 hours using the inverted motorized epifluorescence time lapse Leica DMI 6000- microscope. The normalization of the invasive area to the aggregate size at 24 hours was used based on the technical limitations of keeping the cells inside the time-lapse incubator for the period of 72 hours. All measurements were taken using the ImageJ software (U.S National Institutes of Health). More than seven aggregates were imaged per cell line in each experiment in three or two independent experiments.

**In vivo chorioallantoic membrane (CAM) invasion**

In vivo invasion activity of parental, ∆*MSLN* and rescue OVCAR8 cells was assessed by the CAM assay. Fertilized chick (Gallus gallus) eggs (6 per group/per experiment, in a total of 3 independent experiments) obtained from commercial sources were incubated horizontally at 37.8 °C in a humidified atmosphere and referred to embryonic day (E). On E3 a square window was opened in the shell after removal of 1.5–2 mL of albumen to allow detachment of the developing CAM. The window was sealed with adhesive tape and the eggs returned to the incubator. At E10, 1 × 10^6^ cells of each cell line were loaded inside a silicon ring under sterile conditions. The eggs were re-sealed and returned to the incubator for an additional 3 days. After removing the ring, the CAM was excised from the embryos, photographed ex ovo under a stereoscope, at 20× magnification (Olympus, SZX16 coupled with a DP71 camera). CAMs bearing the tumors were fixed in 10% neutral-buffered formalin and paraffin-embedded for slide sections. The analysis of cell invasion was performed in a blind fashion manner by two independent observers (LD and SR) and slides were scored as previously described (8), with minor alterations: Score 1- without tumour cell invasion of CAM; score 2- tumor cells are tight together forming a compact mass. The invasion front (area where tumor cells touch the CAM mesenchyme) is clearly defined as an encapsulated -like structure; score 3- tumor cells are oriented towards the invasion front and it is possible to observe single cells or small clusters of cells disconnected from the invasive front.

**MTT cell proliferation/viability assay**

To evaluate the proliferation/viability, cells were seeded at the density of 750 to 2000 cells/well, into 96 well-plates and incubated up to 7 days. At each time point, MTT dye (Sigma-Aldrich) was added at a final concentration of 500µg/ml and incubated for 3 hours. After the incubation time, supernatant was removed and 200µl of DMSO was added to dissolve the crystals. The absorbance at 570nm was measured with Synergy H1 Hybrid Reader (Biotek). The measurement was performed in three independent experiments, each performed in quadruplicate.

**Anoikis resistance assay**

To evaluate anoikis resistance, cells were seeded at the density of 1x10^6^ cells/well into 6 well poly-HEMA coated plates (Sigma-Aldrich) and incubated up to 11 days. At each time point cells were harvested, washed, and dissociated by trypsin. Cell suspensions were then incubated for 2 minutes with DAPI at the final concentration of 1µg/ml and immediately analyzed by flow cytometry using the FACS Canto II (BD Bioscience) and data was analyzed by FlowJo software v10.0.7 (Tree Star Inc). All experiments were performed in triplicate in three independent experiments.

**Anchorage independent cell growth assay**

To evaluate the anchorage-independent cell growth, cells were resuspended in culture medium containing 0.3% of agar (Sigma-Aldrich). The mixed cell-agar suspensions (1000cells/well) were immediately plated into 6-well plates coated with 1% agar in culture medium. After 15 days of culture, colonies were stained with crystal violet (Sigma-Aldrich) and counted using the ImageJ software (U.S. National Institutes of Health). All experiments were performed in triplicate in three independent experiments.

**Mesothelial clearance assays**

MeT5A cells stably expressing EGFP or mCherry protein were seeded into 8-well chamber (Ibidi) coated with 10µg/ml of collagen type I (Millipore) and incubated for 24 hours to form confluent monolayers. In parallel, aggregates of ovarian cancer cells were generated by seeding 300 cells/well into 96-well U bottom poly-HEMA (Sigma-Aldrich) coated plates. After incubating for 24 hours, the aggregates were transferred to the wells containing the MeT5A-EGFP or MeT5A-mCherry monolayers. Using the inverted motorized epifluorescence microscope Leica DMI 6000-time lapse, live cell imaging was performed for 24 hours. To quantify the normalized mesothelial clearance area, the non-fluorescent surface created by the invading ovarian cancer cells aggregate in the EGFP or mCherry mesothelial cell monolayer was measured at 18 hours of co-culture and divided by the area of the aggregate at the initial seeding time. For this assay, parental and ∆*MSLN* OVCAR8 cells were reengineered to stably express dTomato protein by lentivirus transduction using the pUltra-Chili plasmid. All measurements were taken using the ImageJ software (U.S National Institutes of Health). More than seven multicellular aggregates were imaged by each cell line in each experiment in a total of three independent experiments.

**Protein extraction and Western blot analysis**

Cells were lysed for 30 minutes on ice in a lysis buffer containing 50mM Tris-HCL (pH 7.5), 150nM NaCl, 2mM ethylenediaminetetraacetic acid and 1% IGEPAL (Sigma- Aldrich), supplemented with Complete protease inhibitor cocktail (Roche Applied Science), 1mM PMSF and 1mM Na_3_VO_4_. Lysates were centrifuged at 14 000 rpm for 20 minutes at 4°C and the supernatant recovered. Protein concentration was determined using the BCA Protein Assay Reagent (Thermo Fisher Scientific). Protein extracts were loaded and separated using SDS-PAGE followed by blotting onto a polyvinylidene difluoride membrane (Amersham Bioscience). Latter, the membrane was blocked with 5% (w/v) BSA (Sigma- Aldrich) or non-fat milk in TBS-T and incubated with primary antibodies, (Supplementary table S2) diluted in 5% (w/v) BSA or non-fat milk in TBST at 4°C overnight. Next, the membranes were washed in TBS-T and incubated with the corresponding secondary antibodies HRP-conjugated (anti-rabbit or anti-mouse) at 1:10000 (Cell Signaling) in TBS-T for 3 hours at room temperature. Signals were detected using the Super Signal West Dura Extended Substrate (Thermo Fisher Scientific).

**Supplementary figures and tables**

**
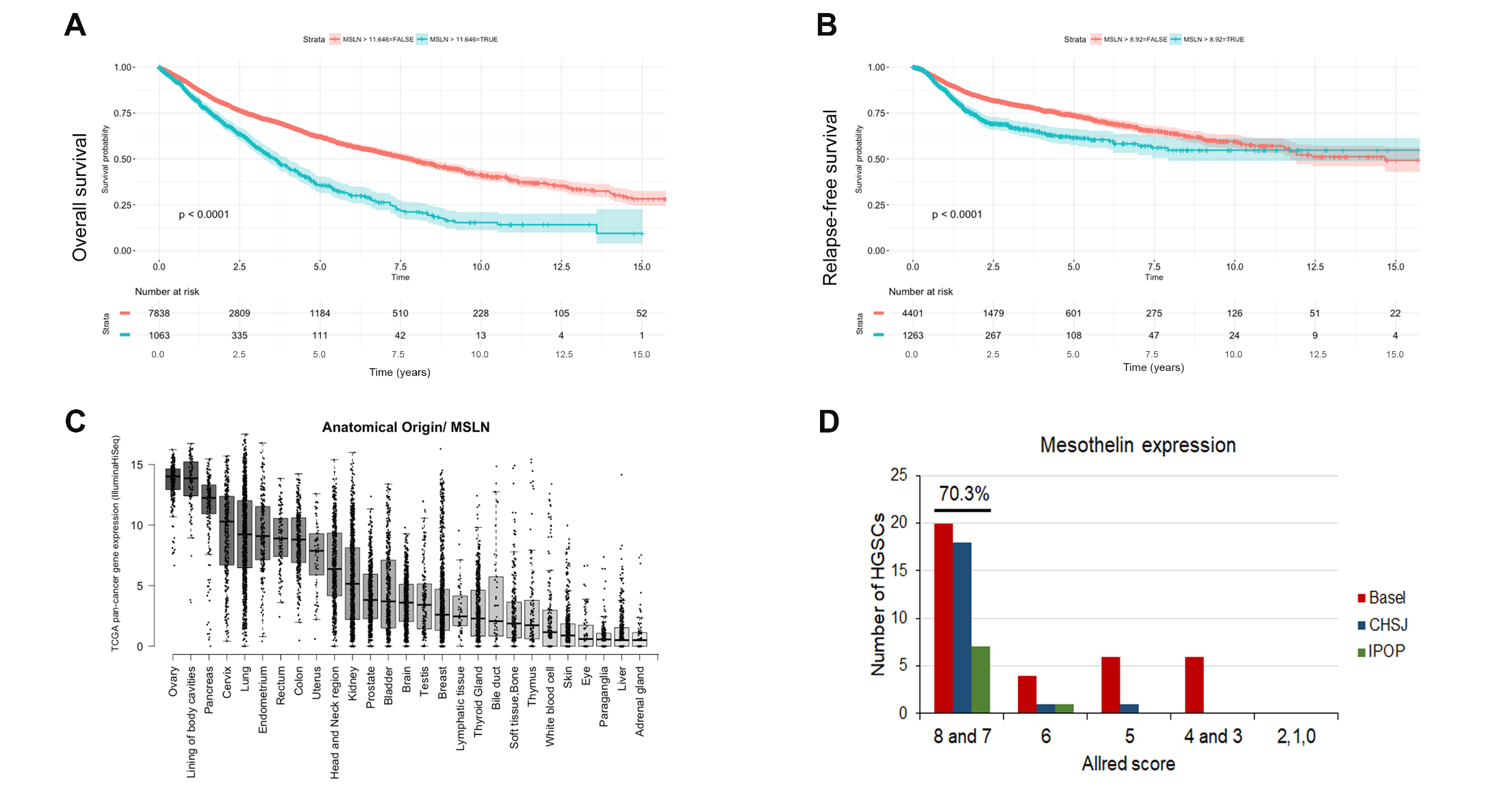
**

**Fig. S1. A, B** Kaplan-Meier method with log-rank test for comparison of survival curves in all TCGA cancer types. The threshold for overall (**A**) and relapse-free survival (**B**) were determined by using conditional inference tree model (R package ‘party’). Survival in patients with high *MSLN* were shorter and relapsed earlier than patients with low *MSLN* expression, *p* values were calculated by log-rank test. **C** Boxplots for *MSLN* expression according to anatomical origin sorted by descending median of *MSLN* expression, among all TCGA cancer types. **D** MSLN immunocytochemistry results for 64 HGSC in three independent series: University Hospital of Basel (n=36), Centro Hospital de São João (CHSJ) (n=20) and Portuguese Oncology Institute of Porto (IPOP) (n=8). The percentage in the bar chart refers to the sum of the number of cases in the three series.

**
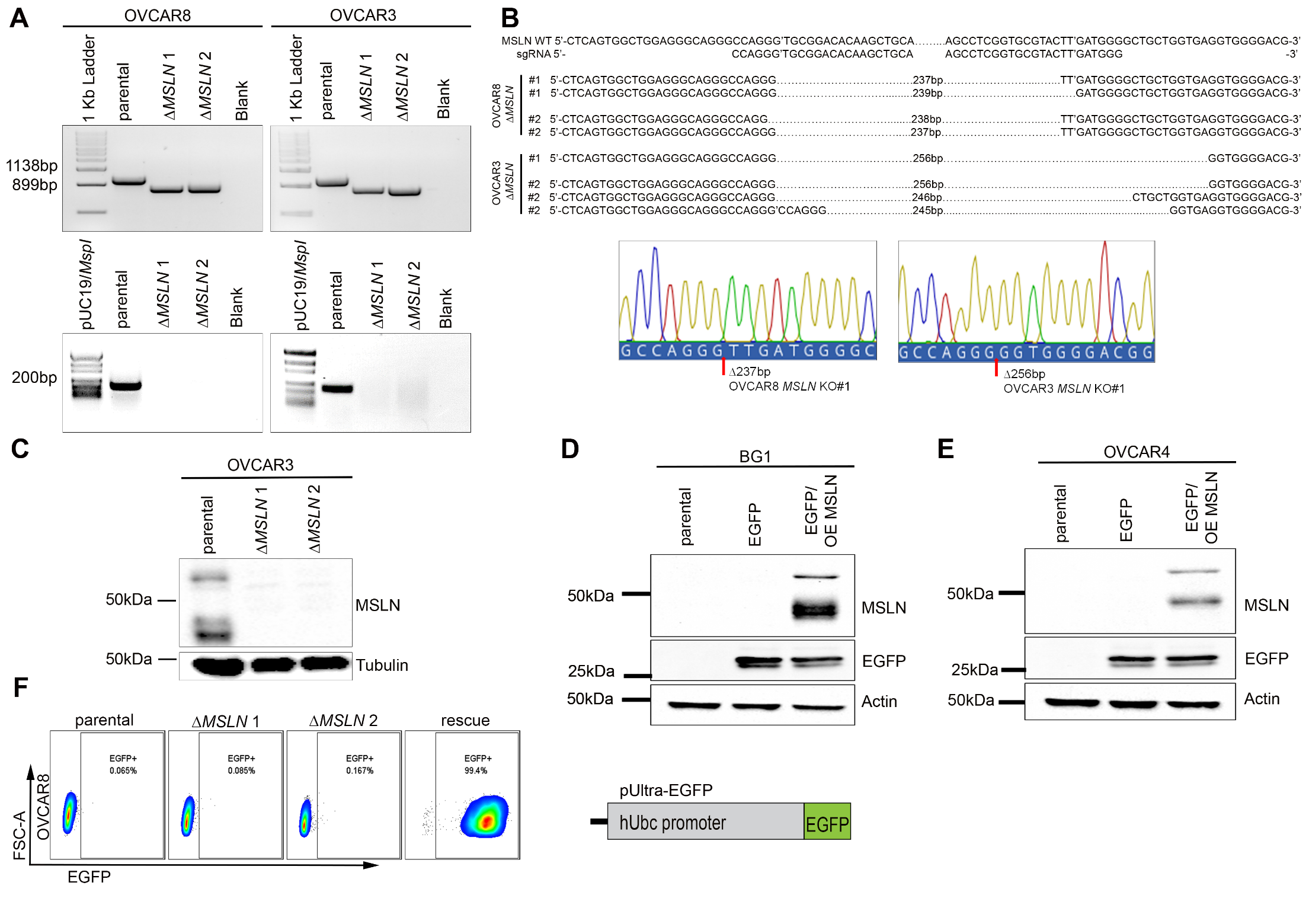
Fig. S2.** **A** Identification of homozygous Δ*MSLN* clones by two genotyping PCRs 1: Deletion PCR (CRISPR_F, CRISPR_R), 1138bp and 899bp, 2: Wild type *MSLN* specific PCR (ORF_F, ORF_R), 200bp. **B** Genomic DNA sequence variation at the CRISPR-*Cas9* active sites in selected OVCAR8 and OVCAR3 Δ*MSLN* clones. **C** Western blot data showing loss of MSLN expression in OVCAR3 Δ*MSLN* clones. **D,** **E** Western blot data showing expression of EGFP in control cells (transduced with pUltra) and MSLN-EGFP expression in overexpressing MSLN (OE MSLN) BG1 (**D**) and OVCAR4 (**E**) cells (transduced with pUltra-MSLN). Depiction of the construct used to establish cell lines with stable expression of EGFP. **F** Flow-cytometry data showing expression of EGFP in Δ*MSLN* rescue OVCAR8 cells after lentivirus transduction of pUltraEGFP-MSLN.


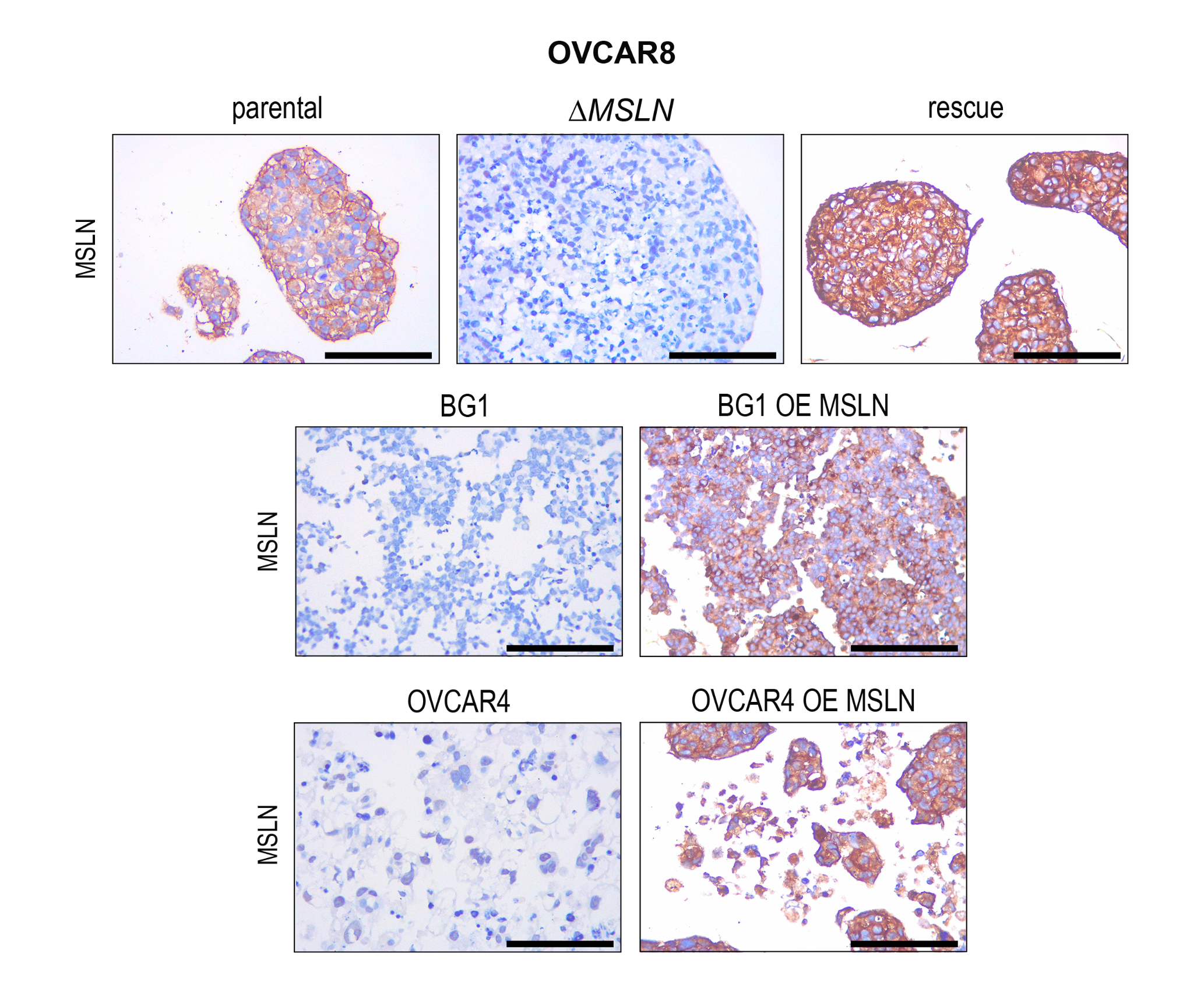


**Fig. S3.** Representative immunocytochemistry images for MSLN in OVCAR8 (parental, ∆*MSLN*, and rescue), BG1 and OVCAR4 (parental and OE MSLN) ovarian cancer cell lines. Scale bar 100µm.


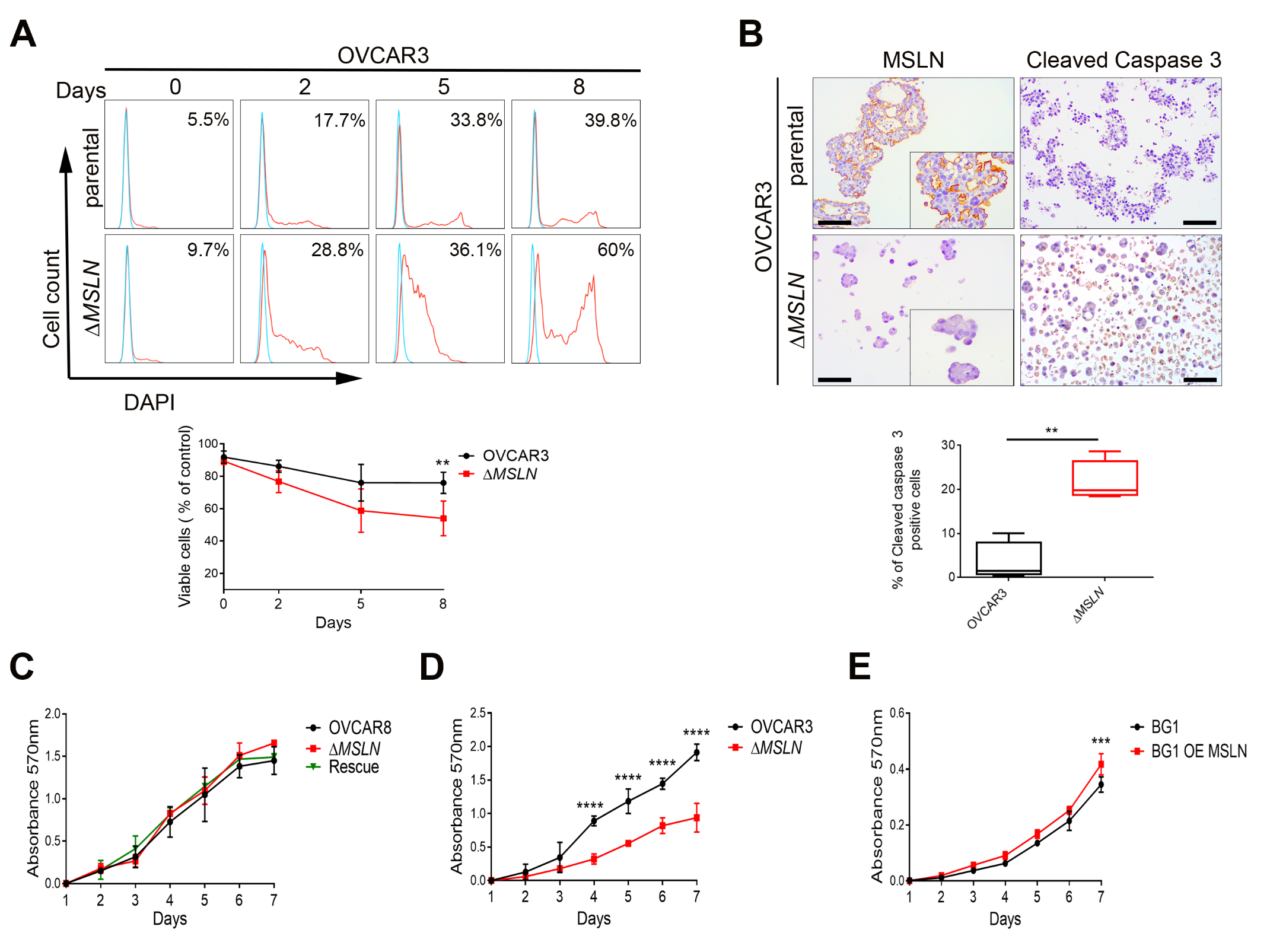


**Fig. S4. A** Histogram for cell-detachment-induced apoptosis (anoikis) for OVCAR3 cells. Unstained or negative control (blue) and percentage of DAPI stained cells (dead cells, red). The percentage in each histogram refers to the DAPI positivity. Corresponding line chart showing the percentage of viable cells–DAPI negative cells, between day 0 and day 8. **B** Representative immunocytochemistry images for cleaved caspase 3 and MSLN of cells cultured for 8 days in low adhesion conditions. Boxplots showing the percentage of cleaved caspase 3 positive cells. Quantification of the percentage of cleaved caspase 3 positive cells was done using QuPath software. **C-E** MTT cell viability and proliferation assay for OVCAR8 (**C**), OVCAR3 (**D**) and BG1 (**E**) cells. Line charts are shown as mean ± SD and boxplots are shown as median and interquartile range of 3 (**A**, **C**, **D** and **E**) or 2 (**B**) independent experiments, *p* values were calculated by two-way ANOVA followed by Sidak’s multiple comparison test (**A**, **D** and **E**) or Turkey`s multiple comparison test (**C**) and unpaired, two-tailed-*t* test (**B**), (**, *p* <0.01, ****, p*<0.001, ****, *p*<0.0001). Scale bar 100µm.

**
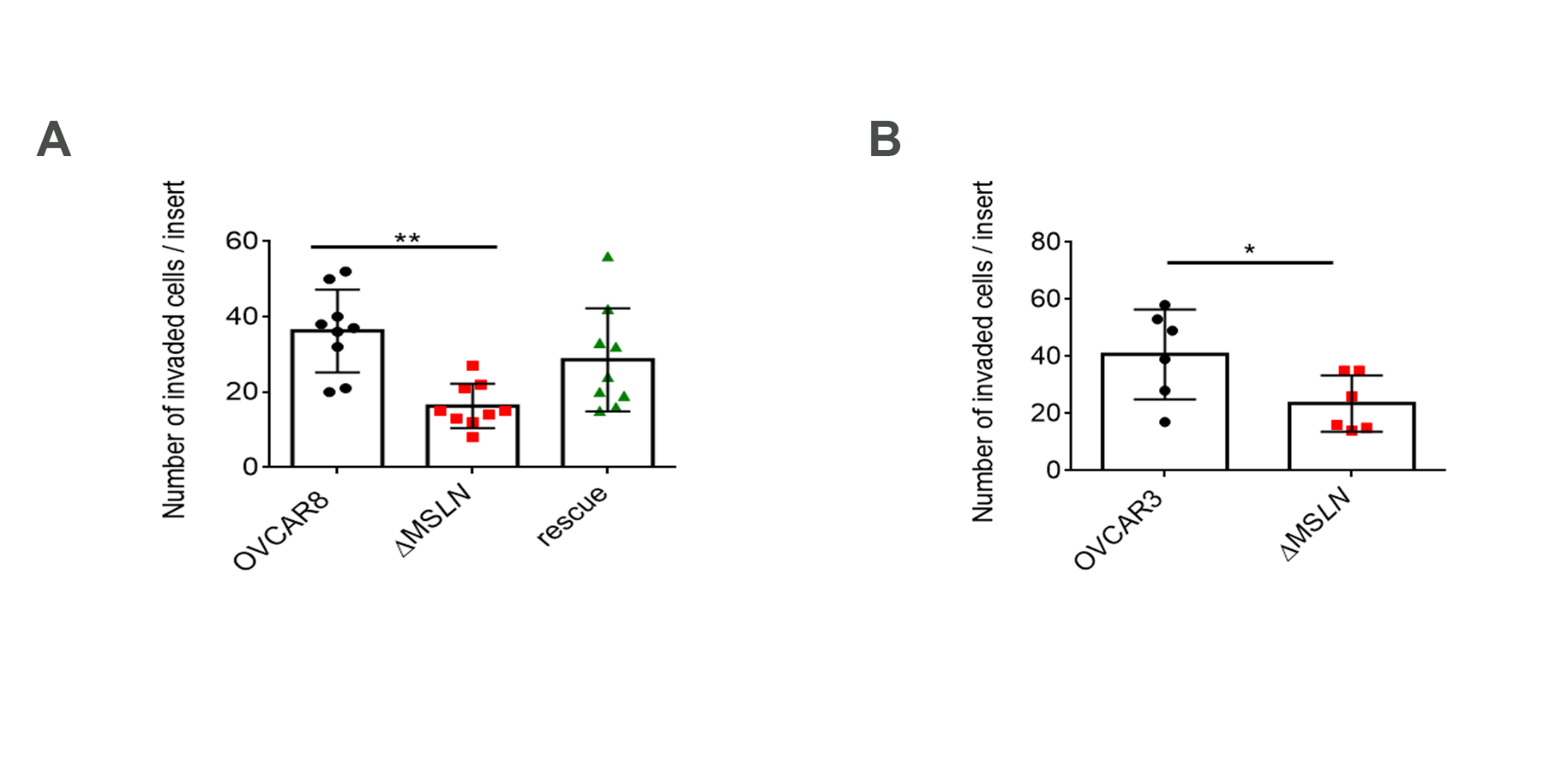
**

**Fig. S5. A**, **B** Quantification of 2D transwell invasion capacity of OVCAR8 (**A**) and OVCAR3 (**B**) cells. Data are shown as mean ± SD of 3 independent experiments done in triplicate (**A**) or duplicate (**B**). *p* values were calculated by one-way ANOVA followed by Turkey’s multiple comparison test (**A**) or unpaired, two-tailed-*t* test (**B**) (*, *p* <0.05, **, *p* <0.01).


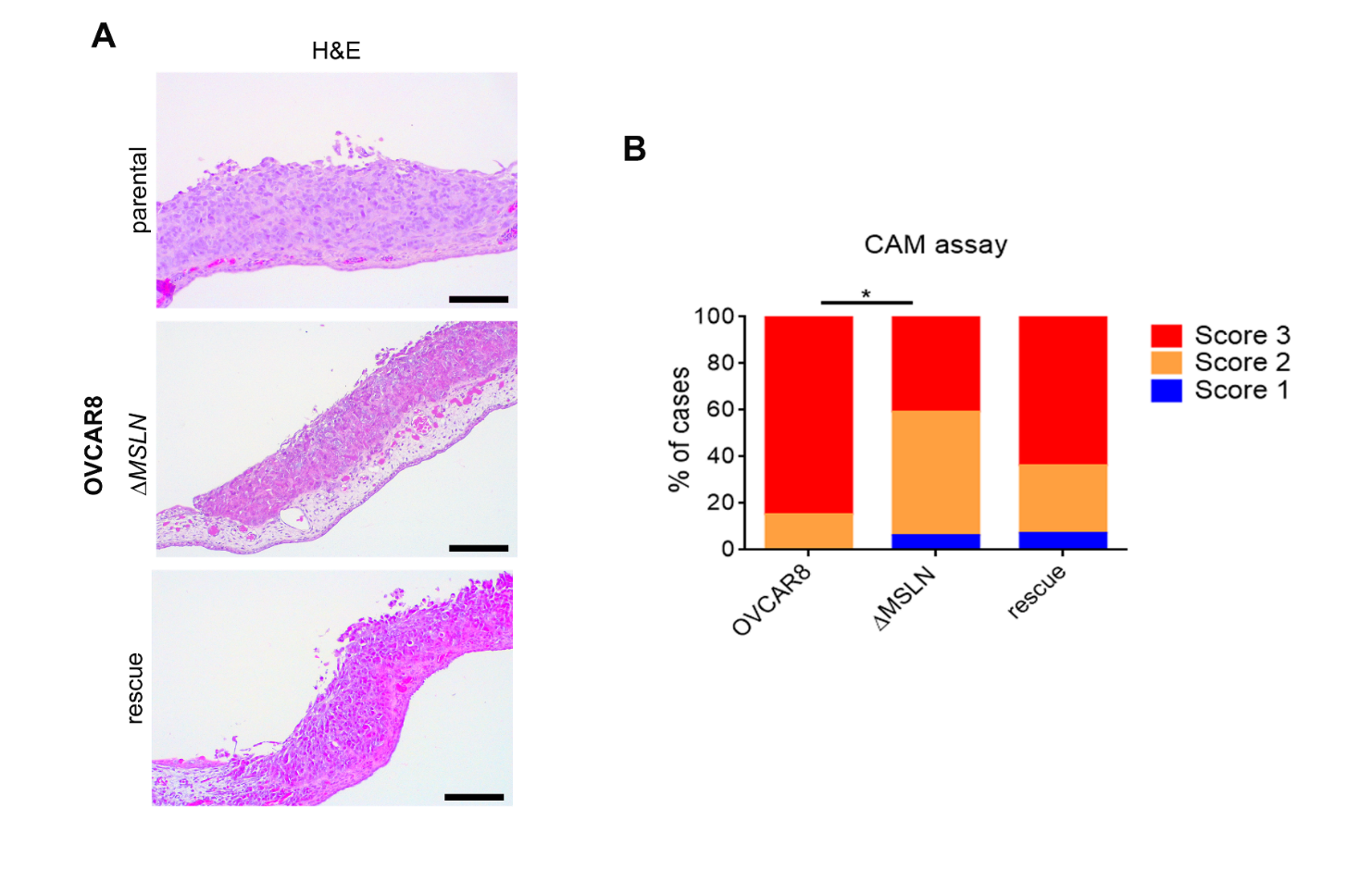


**Fig. S6.** **A** Representative H&E images of the CAM *in vivo* invasion assay for parental, ∆*MSLN* and rescue OVCAR8 cells. Scale bar 100µm. **B** Bar chart showing the percentage of cases in each score for parental, ∆*MSLN* and rescue OVCAR8 cells; Score 1- without tumour cell invasion of CAM; Score 2- tumor cells are tight together forming a compact mass. The invasion front (area where tumor cells touch the CAM mesenchyme) is clearly defined as an encapsulated -like structure; Score 3- tumor cells are oriented towards the invasion front and it is possible to observe single cells or small clusters of cells disconnected from the invasive front. Data are shown as percentage of cases in each score in 3 independent experiments using 6 eggs per condition/experiment, *p* values were calculated by two-way ANOVA followed by Turkey’s multiple comparison test (**B**) (*, *p* <0.05).


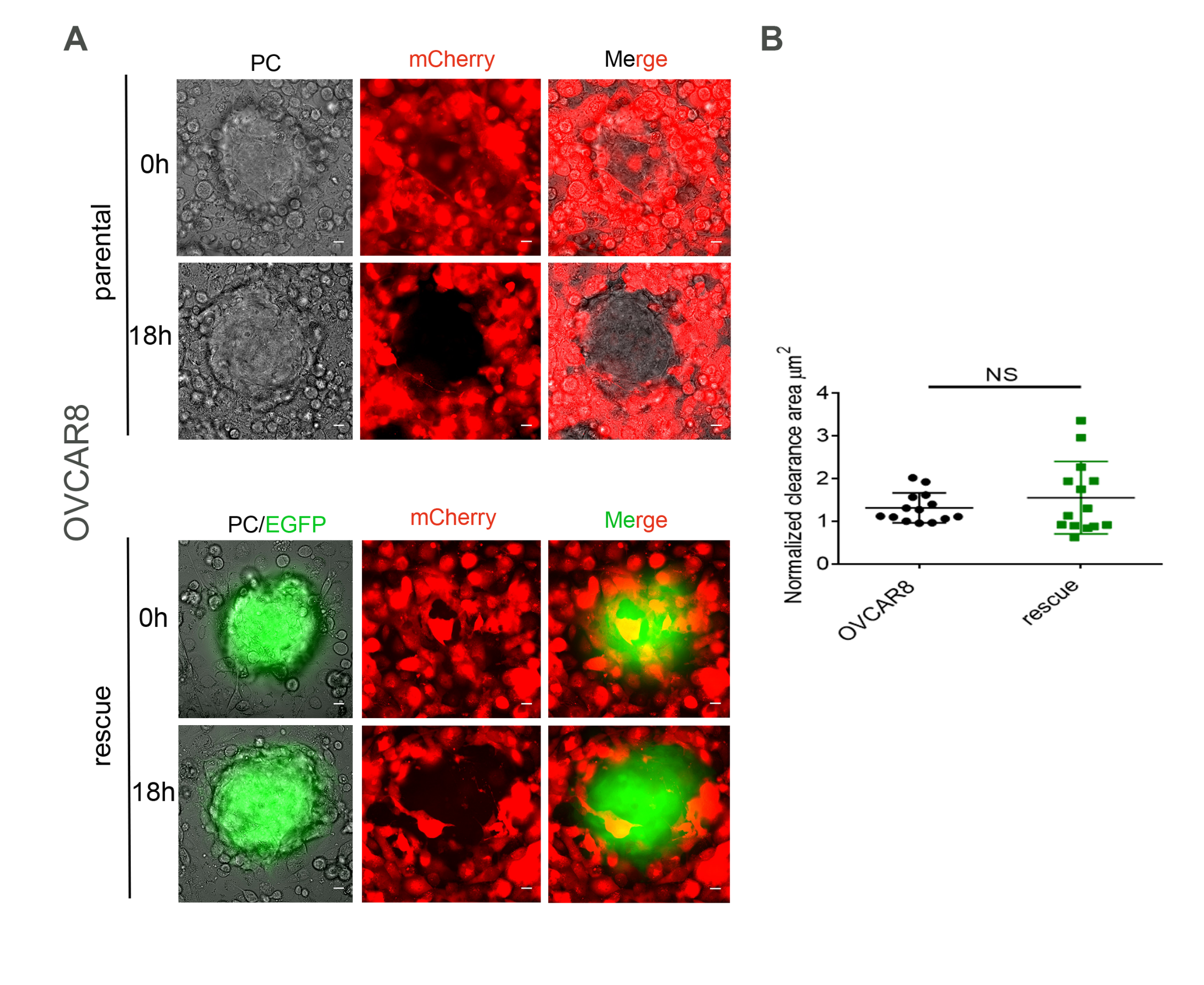


**Fig. S7**. **A** Representative images of mesothelial clearance assay for parental and rescue OVCAR8 cells. **B** Dot blot showing the normalized clearance area for parental and rescue OVCAR8 cells. Data are shown as mean ± SD of 2 independent experiments, more than 6 aggregates were used per condition/experiment. *p* value was calculated by unpaired, two-tailed-*t* test. PC, phase contrast. NS, not significant.

**
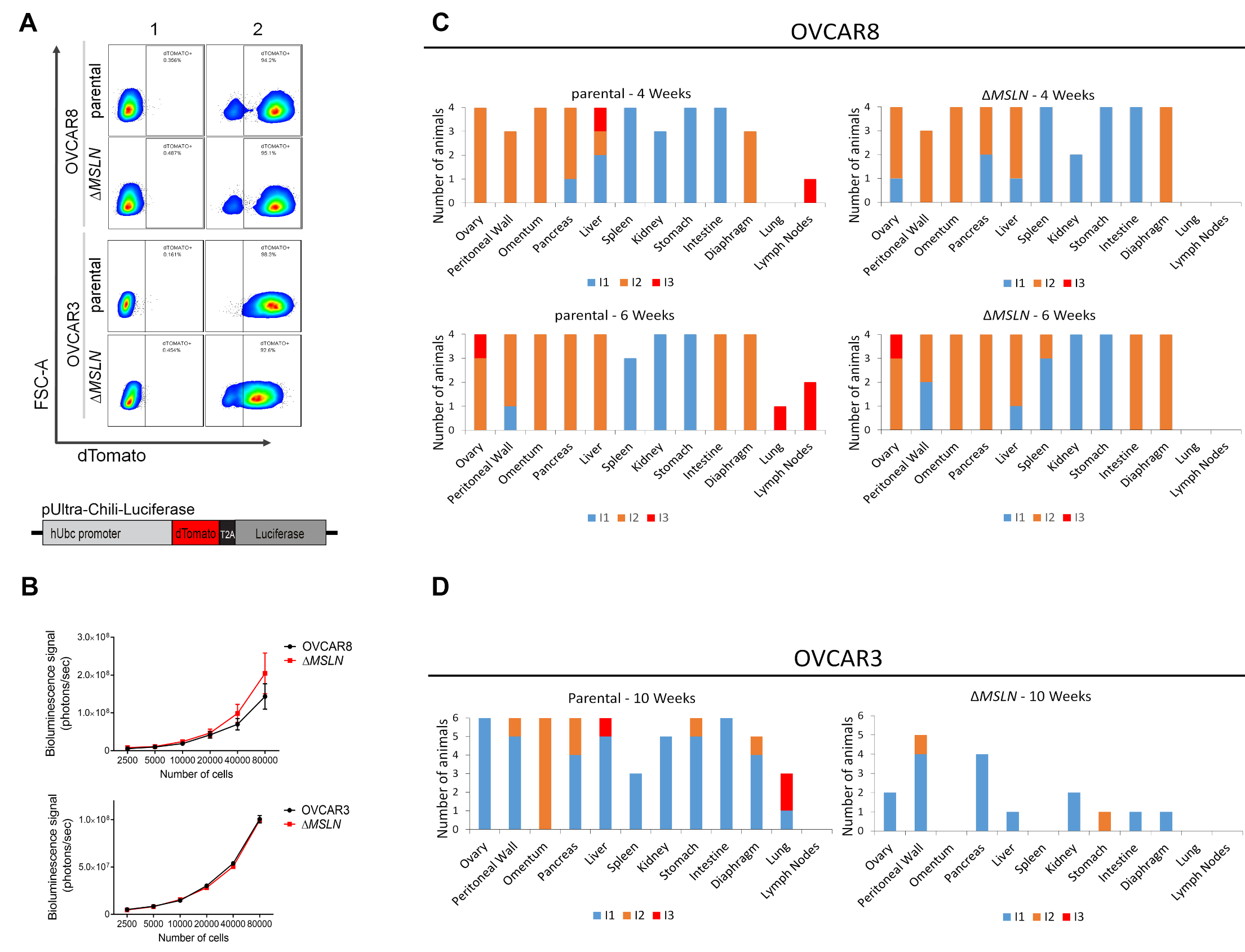
Fig. S8. A** Characterization of OVCAR8 and OVCAR3 luciferase labeled cells by flow cytometry. 1- Untransduced/control cells; 2- Transduced and sorted cells; Depiction of the construct used to establish cell lines with stable expression of dTomato and Luciferase. **B** Line chart showing the quantification of bioluminescence signals for OVCAR8 and OVCAR3 luciferase labeled cells. Data are shown as mean ± SD 3 independent experiments. **C** and **D,** Bar charts showing the number of animals with metastases/invasion for OVCAR8 (n=8 per group) and OVCAR3 (n=6 per group) xenografts. OVCAR8 xenografts were euthanized in two different time points, 4 and 6 weeks after the intraperitoneal injection. OVCAR3 xenografts were euthanized 10 weeks after the intraperitoneal injection. I1, tumour cells on the surface of the organ without capsule invasion; I2, tumour cells with organ capsule invasion; I3, organ parenchyma with presence of tumour cells or distance metastases.

**
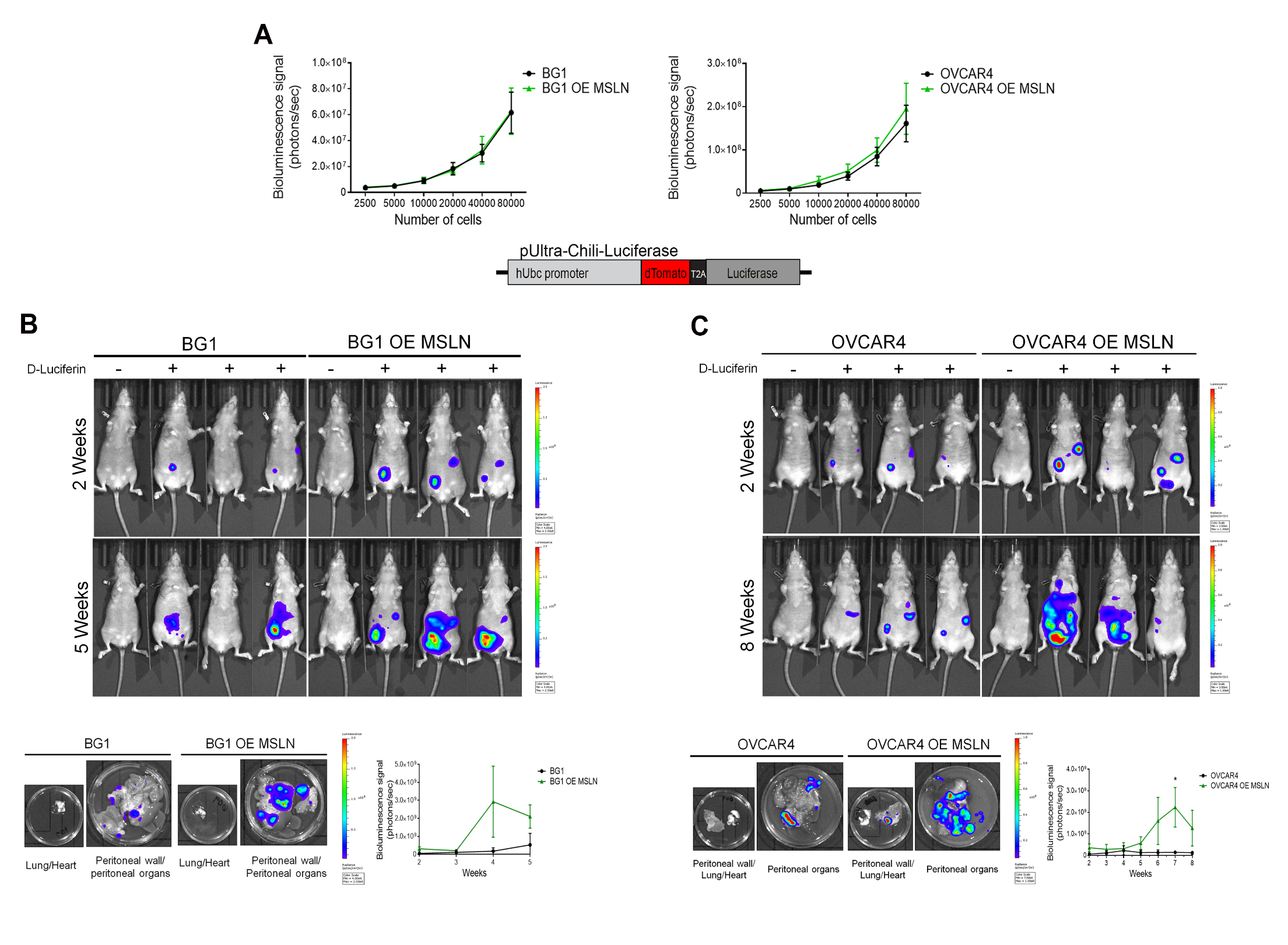
**

**Fig. S9.** **A** Line chart showing the quantification of bioluminescence signals for BG1 and OVCAR4 luciferase labeled cells. Data are shown as mean ± SD 3 independent experiments. Depiction of the construct used to establish cell lines with stable expression of dTomato and Luciferase. **B** and **C,** Representative bioluminescence images for BG1 (n=3 per group) (**B**) and OVCAR4 (n=3 per group) (**C**) xenografts. Bioluminescence signals in the peritoneal organs, peritoneal wall and lung/heart are also shown. Line chart showing the quantification of luciferase signals (photons/sec) between week 2 and week 5 (**B**) or week 8 (**C**) after intraperitoneal injection of BG1 and OVCAR4 luciferase labeled cells. Bioluminescence signals were measure continuously at 1 minute intervals 5 minutes after subcutaneous injection of 100µl of D-Luciferin at 20mg/ml. Data are shown as mean ± SD and *p* values were calculated by two-way ANOVA followed by Sidak’s multiple comparison test (**A**, **B** and **C**). (*, *p* <0.05).

**
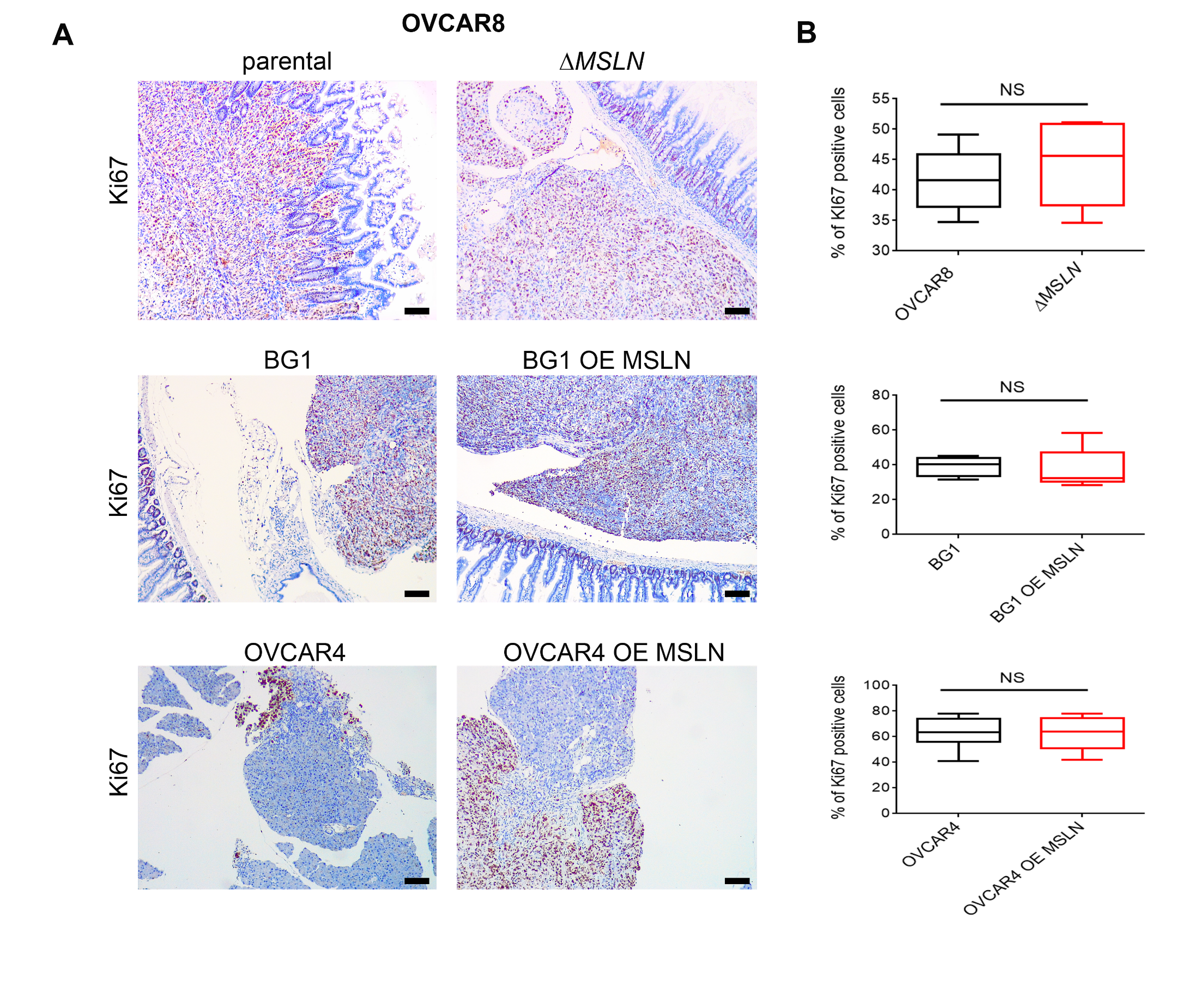
Fig. S10. A** Representative immunocytochemistry images for Ki67 in peritoneal implants of OVCAR8, BG1 and OVCAR4 xenografts. Scale bar 100µm. **B** Boxplots showing the percentage of Ki67 positive cells. Boxplots are shown as median and interquartile range of 6 pictures taken from 3 different mice. *p* values were calculated by unpaired, two-tailed-*t* test. NS, not significant.

**
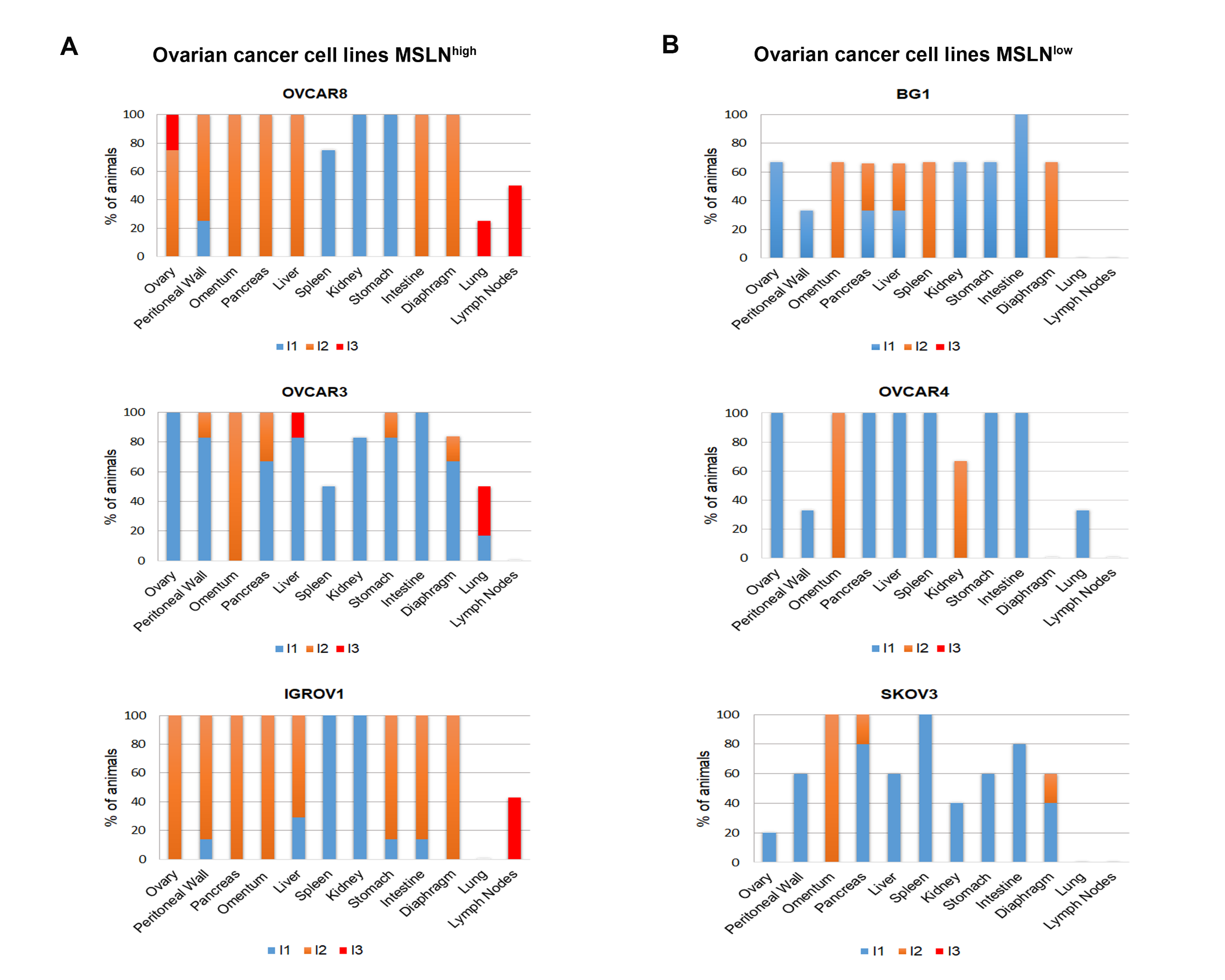
Fig. S11.** **A,** **B** Bar charts showing the percentage of animals with metastases/invasion for xenografts established with ovarian cancer cell lines with high MSLN expression OVCAR8 (n=4), OVCAR3 (n=6) and IGROV1 (n=8) (**A**), and low MSLN expression BG1 (n=3), OVCAR4 (n=3) and SKOV3 (n=5) (**B**). I1, tumour cells on the surface of the organ without capsule invasion; I2, tumour cells with organ capsule invasion; I3, organ parenchyma with presence of tumour cells or distance metastases.

**

**

**Fig.S12.** Western blot data, showing the total expression and activation (phosphorylation) of ERK1/2 and total expression of MMP7 in ovarian cancer cell lines manipulated for MSLN expression. GAPDH was used as a loading control. All experiments were performed in duplicate and a representative Western blot analysis is depicted.

**Supplementary Table S1.** Oligonucleotides used in the study.

**Supplementary table S2.** List of antibodies used in the study.

**

**

**Supplementary references**

1. Coelho R*, et al.* Mucins and Truncated O-Glycans Unveil Phenotypic Discrepancies between Serous Ovarian Cancer Cell Lines and Primary Tumours. Int J Mol Sci. 2018; **7**.

2. Bankhead P*, et al.* QuPath: Open source software for digital pathology image analysis. Sci Rep. 2017; **1**, 16878.

3. Allred DC, Harvey JM, Berardo M, Clark GM. Prognostic and predictive factors in breast cancer by immunohistochemical analysis. Mod Pathol. 1998; **2**, 155-168.

4. Ran FA*, et al.* Genome engineering using the CRISPR-Cas9 system. Nat Protoc. 2013; **11**, 2281-2308.

5. Berens EB, Holy JM, Riegel AT, Wellstein A. A Cancer Cell Spheroid Assay to Assess Invasion in a 3D Setting. Journal of visualized experiments : JoVE. 2015; **105**.

6. Vinci M, Box C, Eccles SA. Three-dimensional (3D) tumor spheroid invasion assay. Journal of visualized experiments : JoVE. 2015; **99**, e52686.

7. Benton G, Arnaoutova I, George J, Kleinman HK, Koblinski J. Matrigel: from discovery and ECM mimicry to assays and models for cancer research. Advanced drug delivery reviews. 2014, 3-18.

8. Ferreira LB*, et al.* Osteopontin-a splice variant is overexpressed in papillary thyroid carcinoma and modulates invasive behavior. Oncotarget. 2016; **32**, 52003-52016.
